# Integrated proteogenomic characterization of medullary thyroid carcinoma

**DOI:** 10.1038/s41421-022-00479-y

**Published:** 2022-11-08

**Authors:** Xiao Shi, Yaoting Sun, Cenkai Shen, Yan Zhang, Rongliang Shi, Fan Zhang, Tian Liao, Guojun Lv, Zhengcai Zhu, Lianghe Jiao, Peng Li, Tiansheng Xu, Ning Qu, Naisi Huang, Jiaqian Hu, Tingting Zhang, Yanzi Gu, Guangqi Qin, Haixia Guan, Weilin Pu, Yuan Li, Xiang Geng, Yan Zhang, Tongzhen Chen, Shenglin Huang, Zhikang Zhang, Shuting Ge, Wu Wang, Weibo Xu, Pengcheng Yu, Zhongwu Lu, Yulong Wang, Liang Guo, Yu Wang, Tiannan Guo, Qinghai Ji, Wenjun Wei

**Affiliations:** 1 Department of Head and Neck Surgery, Fudan University Shanghai Cancer Center; Department of Oncology, Shanghai Medical College, Fudan University, Shanghai, China; 2 Westlake Laboratory of Life Sciences and Biomedicine, Key Laboratory of Structural Biology of Zhejiang Province, School of Life Sciences; Institute of Basic Medical Sciences, Westlake Institute for Advanced Study; Research Center for Industries of the Future, Westlake University, Hangzhou, Zhejiang, China; 3 Department of Head and Neck Surgery, Institute of Cancer Research and Basic Medical Sciences of Chinese Academy of Sciences, Cancer Hospital of University of Chinese Academy of Sciences, Zhejiang Cancer Hospital, Hangzhou, Zhejiang, China; 4 Shanghai OE Biotech Co., Ltd, Shanghai, China; 5 Department of General Surgery, Changzhou Second People’s Hospital, Changzhou, Jiangsu China; 6 Department of Thyroid and Breast Surgery, Taizhou People’s Hospital, Taizhou, Jiangsu China; 7 Department of Thyroid and Parathyroid Surgery, Peking University Shenzhen Hospital, Shenzhen Peking University-The Hong Kong University of Science and Technology Medical Centre, Shenzhen, Guangdong, China; 8 Biobank, Fudan University Shanghai Cancer Center, Shanghai, China; 9 Department of Endocrinology, Guangdong Provincial People’s Hospital, Guangdong Academy of Medical Sciences; The Second School of Clinical Medicine, Southern Medical University, Guangzhou, Guangdong, China; 10 State Key Laboratory of Genetic Engineering, Collaborative Innovation Center for Genetics and Development, School of Life Sciences; Human Phenome Institute, Fudan University, Shanghai, China; 11 Department of Pathology, Fudan University Shanghai Cancer Center; Department of Oncology, Shanghai Medical College, Fudan University, Shanghai, China; 12 Department of Integrative Oncology, Fudan University Shanghai Cancer Center; Shanghai Key Laboratory of Medical Epigenetics, International Co-laboratory of Medical Epigenetics and Metabolism (Ministry of Science and Technology), Institutes of Biomedical Sciences, Fudan University, Shanghai, China; 13 Shanghai Luming Biological Technology Co., Ltd., Shanghai, China

**Keywords:** Thyroid cancer, Neuroendocrine cancer, Cancer genomics, Tumour heterogeneity

## Abstract

Medullary thyroid carcinoma (MTC) is a rare neuroendocrine malignancy derived from parafollicular cells (C cells) of the thyroid. Here we presented a comprehensive multi-omics landscape of 102 MTCs through whole-exome sequencing, RNA sequencing, DNA methylation array, proteomic and phosphoproteomic profiling. Integrated analyses identified *BRAF* and *NF1* as novel driver genes in addition to the well-characterized *RET* and *RAS* proto-oncogenes. Proteome-based stratification of MTCs revealed three molecularly heterogeneous subtypes named as: (1) Metabolic, (2) Basal and (3) Mesenchymal, which are distinct in genetic drivers, epigenetic modification profiles, clinicopathologic factors and clinical outcomes. Furthermore, we explored putative therapeutic targets of each proteomic subtype, and found that two tenascin family members TNC/TNXB might serve as potential prognostic biomarkers for MTC. Collectively, our study expands the knowledge of MTC biology and therapeutic vulnerabilities, which may serve as an important resource for future investigation on this malignancy.

## Introduction

Medullary thyroid cancer (MTC) is a rare neuroendocrine malignancy originating from parafollicular cells (C cells) of the thyroid. Although MTC comprises < 3% of all thyroid neoplasms, it is disproportionally responsible for ~13% of mortalities^1,2^. Compared with differentiated thyroid cancer, MTC usually shows a more aggressive clinical course and a stronger inherited tendency. Approximately 25% of MTCs occur in a hereditary form as multiple endocrine neoplasia type 2 A (MEN2A), MEN2B or familial MTC (FMTC, a variant of MEN2A), virtually all of which are caused by germline mutations in the *RET* proto-oncogene. On the other hand, the remaining ~75% of MTCs exhibit a sporadic pattern, with half of which harbor somatic *RET* alterations followed by mutually exclusive *RAS* mutations^3^. However, there is still a paucity of knowledge regarding MTC genome, with *RET* and *RAS* being the only commonly recognized driver genes up to date^4^. Thus, the “dark matter” MTC samples without any previously known driver events, albeit at a low proportion, require a deeper investigation.

Despite a favorable outcome at early stage, advanced MTCs are incurable with limited treatment options. Chemotherapy and radiotherapy seem to offer little clinical benefit, while two Food and Drug Administration (FDA)-approved agents, vandetanib and cabozantinib, bring moderate responses with significant toxicities^5^. Recently, selective RET inhibitors pralsetinib (BLU-667) and selpercatinib (LOXO-292) have achieved an impressive efficacy in *RET*-altered tumors^6,7^. However, *RET*-wild-type MTCs are not included in their indications and moreover, acquired resistance to these two drugs can also emerge in *RET*-mutant cases^8,9^. Thus, endeavors to develop more precise strategies are still in urgent need, for which clarifying the connections between genetic alterations and tumor phenotypes is a major focus. Proteogenomics are now increasingly used to decipher comprehensive landscape from genotype to phenotype in different cancers^10,11^. Unfortunately, there is still a lack of multi-omics studies of MTC up to now, resulting in a paucity of available data for further research.

In recent years, Fudan University Shanghai Cancer Center has made a massive effort to establish a large biobank of samples from MTC patients in collaboration with tertiary hospitals in the Oriental Thyroid Tumor Specialist Alliance (OTTA) consortium. In the present study, based on this large Chinese multicenter platform, we performed a comprehensive genomic, transcriptomic, epigenomic, proteomic and phosphoproteomic analyses for a large number of patients diagnosed with this rare malignancy. Collectively, our study provides a systematic multi-omics resource of MTC that leads to functional insights of genomic aberrations and facilitates exploration of proteogenomics-based treatment.

## Results

### Patient cohort

Based on the criteria listed in Supplementary Fig. S1 and Materials and Methods, our final study cohort included 102 MTC patients from five Chinese tertiary hospitals in the OTTA consortium. For the entire cohort, the median age at initial surgery was 50 years old and there were no apparent discrepancies between two genders (male: 51%; female: 49%). Twenty-five patients (24.5%) were classified as MEN2A (*n* = 23, including FMTC) or MEN2B (*n* = 2) based on family history, clinical manifestation, and germline *RET* mutations (see below). During the follow-up period, 25 (24.5%) and 54 (52.9%) patients experienced structural recurrence/persistent disease (SR/SPD) or biochemical recurrence/persistent disease (BcR/BcPD), respectively. A total of 15 (14.7%) patients initially had or eventually developed into advanced disease (including both locally advanced disease or distant metastasis). Detailed patient information is summarized in Supplementary Table S1.

### Overview of the proteogenomic profiling of Chinese MTCs

**Fig. 1:**
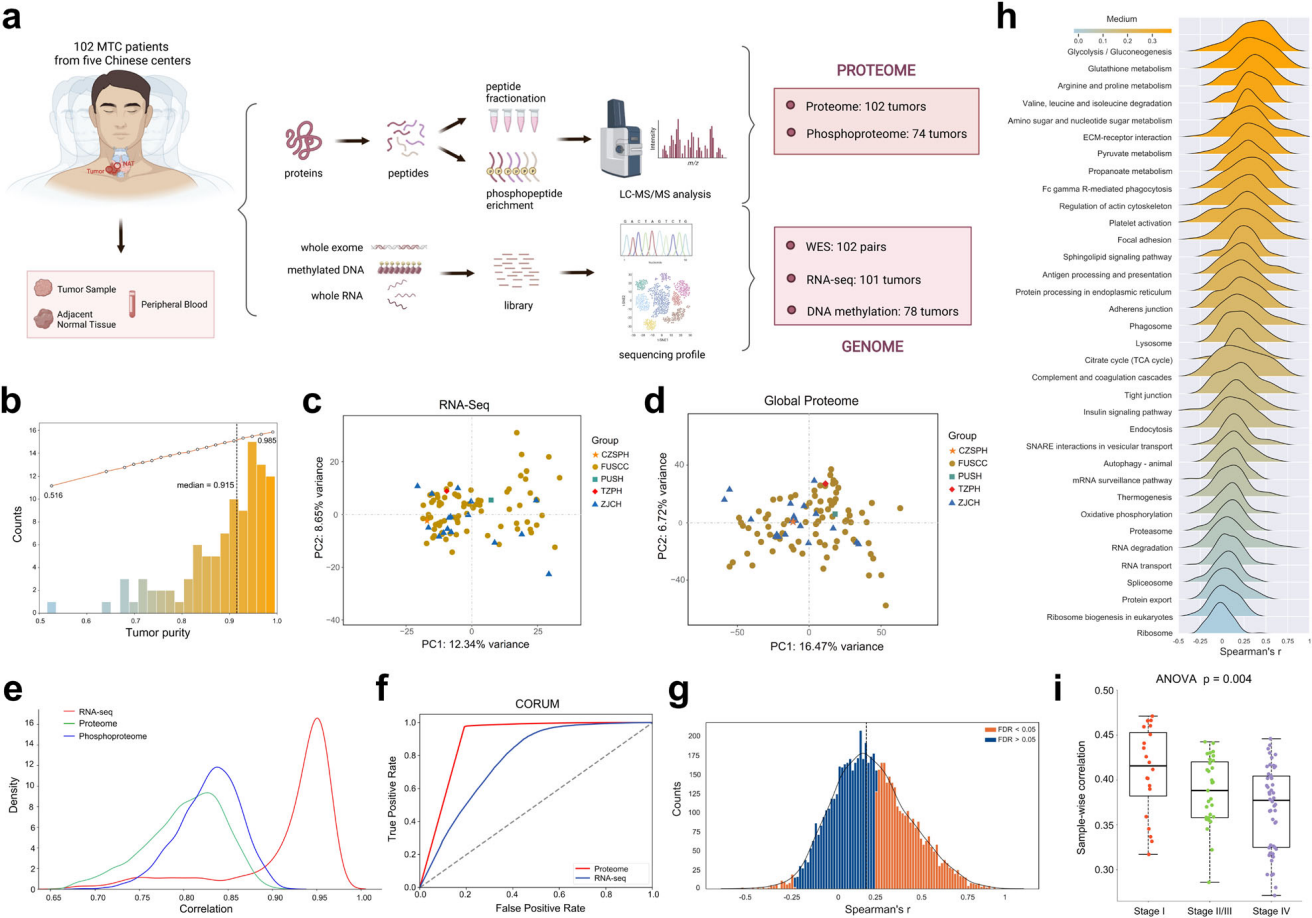
Proteogenomic summary of the study. **a** Schematic overview of the experimental design and number of samples for WES, RNA-Seq, DNA methylation array, proteomics and phosphoproteomics analyses. **b** Histogram showing tumor purity of samples (median: 91.5%) in our study cohort, as estimated by ESTIMATE algorithm using RNA-Seq data. **c**, **d** Principal-component analysis plots of RNA-Seq (**c**) and global proteome (**d**) data across the five contributing centers in our study. **e** Density plots showing the distribution of inter-sample Pearson’s correlation coefficient (a given tumor vs others) of RNA-Seq (red line), global proteome (green line) and phosphoproteome data (blue line) across samples in our study cohort, respectively. **f** Area under the receiver operating characteristic curves (AUROC) for prediction of all 4274 protein complex members recorded in the CORUM database (version 3.0) using 54,162,592 RNA–RNA pairs in RNA-Seq data (blue line) and 21,822,921 protein–protein pairs in global proteome data (red line). **g** Histogram showing gene-wise mRNA–protein Spearman’s correlations among the 6454 mRNA–protein pairs. **h** KEGG pathway enrichment for higher or lower gene-wise mRNA–protein correlations (Benjamini–Hochberg FDR < 0.05). **i** Comparison of sample-wise mRNA–protein correlations across tumors with different TNM staging. *P*-values are calculated with ANOVA test. For the boxplot: line in the box indicates the median; box borders correspond with the first and third quartiles (25th and 75th percentiles); whiskers extend 1.5 times the interquartile range; outlier data are shown as dots. See also Supplementary Tables S1, S2.

In total, whole-exome sequencing (WES) was performed on matched tumor–normal (blood or normal thyroid tissue) pairs from all 102 patients in our cohort, while global proteomics and RNA sequencing (RNA-Seq) were performed on tumor samples from 102 and 101 patients, respectively. Due to the small volume of most MTC tumors (especially those ≤ 1 cm), 78 and 74 patients had sufficient DNA and protein for methylation and phosphoproteomic analyses, respectively (Fig. 1a; Supplementary Fig. S2 and Table S4). As revealed by the ESTIMATE algorithm^12^, high tumor purity was predicted with a median value of 91.5%, and no observable batch effects were detected across the five contributing centers in principal component analyses (Fig. 1b–d). Sample correlation analysis across the cohort exhibited greater consistency for RNA-Seq data than proteomic and phosphoproteomic data, while global proteomic data outperformed RNA-Seq data in predicting protein complexes in the CORUM database (Fig. 1e, f). These results illustrated that protein-level regulation could better reflect tumor heterogeneity and gene function compared with transcriptomic profiles.

Similar to previous studies^13,14^, we observed a weak consistency between protein and mRNA abundance with sample-wise and gene-wise median Spearman’s correlation being 0.39 and 0.19, respectively. At the gene level, 43% (*n* = 2775) and 1% (*n* = 83) of the 6454 mRNA–protein pairs displayed significant positive (Spearman’s coefficient > 0, Benjamini–Hochberg false discovery rate (FDR) < 0.05) and negative (Spearman’s coefficient < 0, FDR < 0.05) correlations, respectively (Fig. 1g; Supplementary Table S2). Enrichment analyses showed that genes involved in metabolism-related pathways had stronger correlations, while those involved in oxidative phosphorylation and mRNA/protein processing (including spliceosome, ribosome, proteasome, RNA transport and protein export), mostly featured by the formation of large protein complexes, revealed poorer relevance (Fig. 1h), which was consistent with prior reports of other malignancies^15^. At the sample level, mRNA–protein correlations tended to decline with the increase of TNM staging, indicating overall stronger post-transcriptional regulations in aggressive tumors (Fig. 1i).

### Landscape of driver mutations in Chinese MTCs

**Fig. 2:**
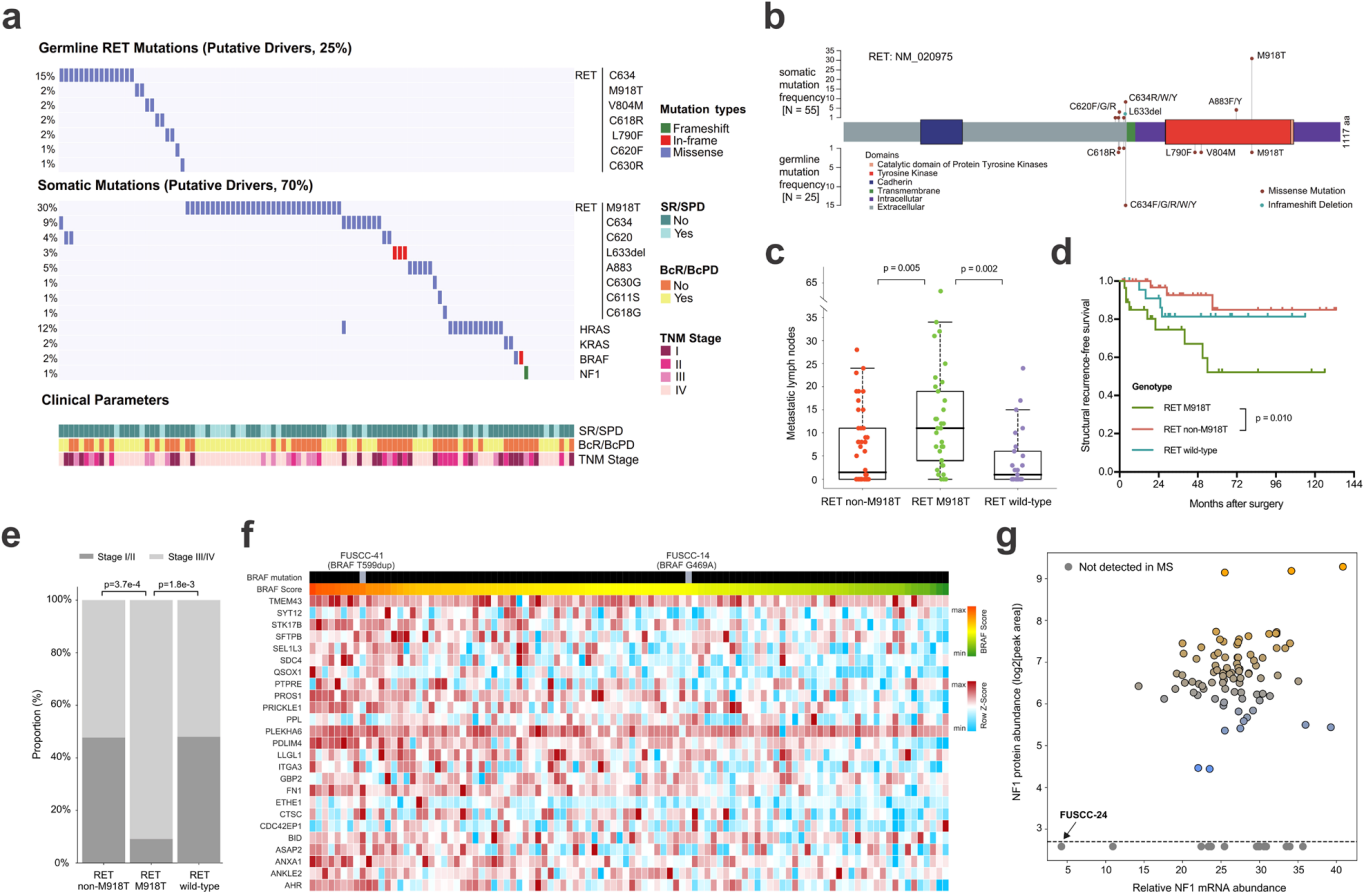
Landscape of driver mutations in the Chinese MTC cohort and their clinicopathologic and downstream Molecular Implications. **a** Profiles of putative driver mutations and associated clinicopathologic features of all the 102 MTC patients. **b** Lollipop plot showing locations of somatic and germline *RET* mutations within the protein sequence. **c**–**e** Comparison of metastatic lymph node counts (**c**, Student’s *t*-test), SRFS (**d**, log-rank test) and TNM staging (**e**, Fisher’s exact test) and between *RET* M918T-mutated, *RET* non-M918T-mutated and *RET* wild-type tumors. For the boxplot in **c**: line in the box indicates the median; box borders correspond with the first and third quartiles (25th and 75th percentiles); whiskers extend 1.5 times the interquartile range; outlier data are shown as dots. **f** Heatmap showing *BRAF* scores and proteomic profiles of 25 related genes across all the 102 MTC tumors. Tumors are arranged in descending order based on *BRAF* score, and the locations of two *BRAF*-mutated tumors are annotated. Protein abundances were displayed in this heatmap after normalization by row Z-score. **g** Scatter plots showing relative *NF1* mRNA abundance (FPKM, *x*-axis) and protein level (manifested as log_2_-transformed XIC peak area, *y*-axis) of all available tumors. See also Supplementary Fig. S3 and Tables S3, S4.

Due to the inherited nature of MEN2, we first analyzed the genomic profiling of 102 paired normal specimens (blood or normal thyroid tissue) to identify hereditary MTCs. A total of 25 patients (24.5%) were regarded as MEN2 based on the presence of pathogenic germline *RET* alterations, with the majority (60%) clustering at codon 634 (Fig. 2a, b). In addition to 23 MEN2A patients (including FMTC), two cases harboring germline *RET*^M918T^ mutation (FUSCC-75, FUSCC-80) were classified as MEN2B due to evidence of mucosal neuroma accompanied with either pheochromocytoma (PHEO) or marfanoid body habitus (Supplementary Table S1).

On the other hand, the remaining 77 individuals were confirmed to be sporadic after excluding MEN2-related syndromes, family history and germline *RET* aberrations. Somatic mutations, called with a strict criterion (Materials and Methods), were then manually reviewed to identify putative driver alterations for these sporadic cases. Among them, somatic *RET* (52/77) and *HRAS* (12/77) mutations occurred in most cases, with M918T and Q61 being the most common hotspots of the two genes, respectively (Supplementary Table S3). Besides, there was striking mutual exclusivity between *RET* and *RAS* mutations (including both *HRAS* and *KRAS*, Fisher’s exact test, *P* = 1.37e–08), corroborating their role as the most dominant drivers in sporadic MTC (Fig. 2a; Supplementary Table S3).

Combining hereditary and sporadic forms together, *RET* mutations were responsible for 75.5% of carcinogenesis in the whole series. Among various mutants, the *RET*^M918T^ mutation portended elevated tumor aggressiveness, including a greater number of metastatic lymph nodes, more advanced TNM staging and a poorer structural recurrence-free survival (SRFS) (Fig. 2c–e).

Previous reports have suggested that MTC lacks commonly recognized mutated driver genes beyond *RET* and *RAS*^4^. In our study, two non-hotspot *BRAF* mutations (p.G469A and p.T599dup, variant allele frequency (VAF) > 40%) were separately detected in two sporadic cases absent of other potential drivers (Fig. 2a; Supplementary Fig. S3a, b and Table S3), both of which have been reported as oncogenic drivers in other cancers^16–20^. Furthermore, to investigate the similarity of downstream signaling between the two non-hotspot *BRAF* mutants and the hotspot *BRAF*^V600E^ mutation, we calculated *BRAF* scores^21^ with shared genes from our global proteomic data. Notably, despite an intermediate *BRAF* score of the *BRAF*^G469A^-mutated tumor, the *BRAF*^T599dup^-mutated tumor lay at the forefront of the cohort (Fig. 2f; Supplementary Table S4). This observation is in accordance with previous findings that the T599dup mutant strongly activates the downstream signaling resembling its neighboring hotspot mutation V600E^18^, while G469A, a class II *BRAF* mutation, leads to a moderate kinase activity^22^. Therefore, albeit at a low frequency, *BRAF* is identified as a novel mutated gene in MTC that may act as a potential driver.

In our cohort, we also noticed a unique MTC case (FUSCC-24) with concomitant neurofibromatosis type 1 (NF1), a multi-system genetic disorder caused by pathogenic alterations in a tumor suppressor gene *NF1*^23^ (Supplementary Fig. S3c). Meanwhile, this 61-year-old male patient also developed a coexisting malignant peripheral nerve sheath tumor (MPNST) in the neck, a malignancy arising from the malignant transformation of neurofibroma, which ultimately caused his death (Supplementary Fig. S3d). For his MTC tumor, despite the lack of previously reported MTC-related alterations, a de novo (acquired in embryo development, causing genetic mosaicism) frameshift mutation c.3338delT in the *NF1* gene was detected with a VAF as high as 76.1%, strongly suggestive of a driver event (Supplementary Fig. S3e and Table S3). Interestingly, this frameshift deletion was also a truncating mutation that turned the codon 1113 into a premature stop codon (UUG → UGA, p.L1113*) (Supplementary Fig. S3f). Nonsense-mediated mRNA decay (NMD) and related protein truncation of tumor suppressor genes are thought to be important mechanisms of truncating mutation-directed cancer initiation and evolution^24^. Consistently, these downstream truncating effects of this *NF1* p.L1113* mutation were laterally confirmed by our RNA-Seq and proteomic data, in which this sample revealed the lowest *NF1* mRNA level and undetectable protein abundance (Fig. 2g), further supporting its pathogenic role. Taken together, our study identifies an *NF1* truncating mutation as a putative driver of MTC oncogenesis by multi-omics data integration.

In summary, combining multi-platform data from WES, RNA-Seq and global proteomics, we confirmed the dominant role of *RET* and *RAS* mutations in MTC and identified potential driver alterations (*BRAF*, *NF1*) that were mutually exclusive to *RET* and *RAS*. Ultimately, we have identified putative cancer drivers in 93/102 MTCs (91.2%) in our cohort, reducing the proportion of “dark matter” cases from 18.3% in a previous study to 8.8% in our present work^25^. It is also worth noting that these driver genes in our cohort are all highlighted in the genomic profiles of pheochromocytoma and paraganglioma in a The Cancer Genome Atlas (TCGA) study^20^, suggesting a degree of etiological similarity in these neural crest-derived neuroendocrine tumors.

### Proteomic profiling delineates molecular subtypes of MTC with genomic, epigenomic and clinical heterogeneity

**Fig. 3:**
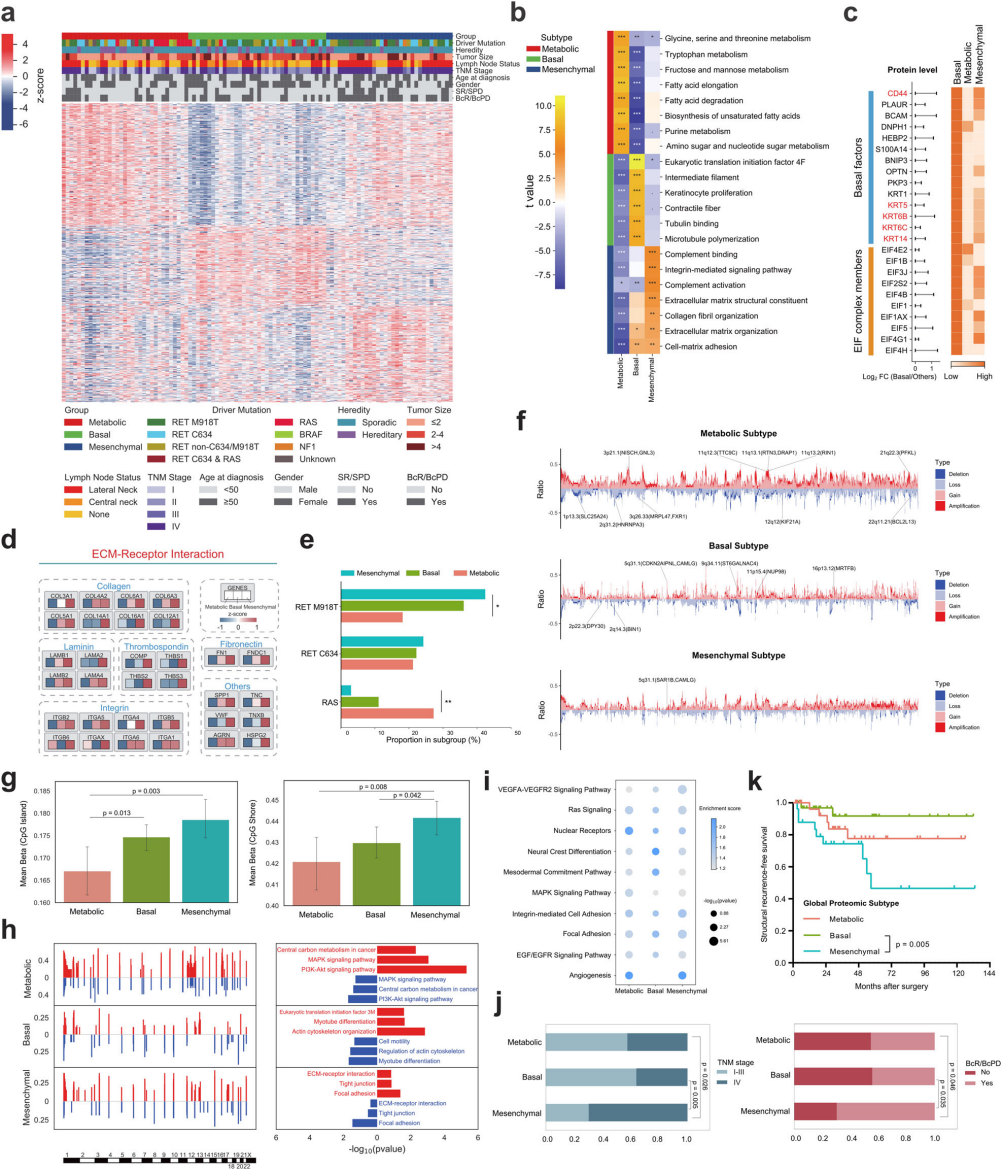
Proteomic subtyping of MTC and their clinical, genomic and epigenomic Correlations. **a** Unsupervised clustering based on the relative abundance of 891 proteins identifies three MTC subtypes: Metabolic (*n* = 33), Basal (*n* = 36), and Mesenchymal (*n* = 33). Clinicopathologic characteristics, prognostic features and driver mutations are annotated above the heatmap with details shown at the bottom. Each column represents a patient sample and rows indicate proteins. Protein abundances were displayed in this heatmap after normalization by row Z-score. **b** Pathway-level analysis of each proteomic subtype shows relative pathway activity (represented as t-value) of cancer hallmark gene sets derived from gene set variation analysis (GSVA). Benjamini–Hochberg FDR values are annotated as follows: ***FDR < 0.001, **FDR < 0.01, *FDR < 0.05. **c** Differential protein abundance of basal factors and eukaryotic initiation factor (EIF) complex members across the three proteomic clusters. The horizontal bars represent the log_2_-transformed FC value of protein abundance between Basal tumors and the other samples. The length of horizontal bars represents the size of log_2_FC (a longer bar corresponds to a larger log_2_FC), while the color scale of the heatmap represents the expression of these proteins in the three subtypes (a deeper color represents a higher protein abundance), which was calculated using the Z-score-transformed average protein abundance within each subtype. **d** Abundance of signature protein families (including collagens, laminins, thrombospondins, integrins, fibronectins and others) involved in the KEGG pathway ECM–Receptor Interaction across the three proteomic subtypes. The value of each protein was calculated using the Z-score-transformed average protein abundance within each subtype. **e** Bar plots showing the proportion of three proteomic subtypes in *RET*^M918T^-mutant, *RET*^C634^-mutant and *RAS*-mutant tumors. *P*-values are calculated with Fisher’s exact test, *P < 0.05. **f** Frequency of SCNA in the three proteomic subtypes. Representative subtype-specific SCNAs and contained genes whose SCNA-revealed *cis*-effect on their cognate proteins were annotated. **g** Histograms comparing the global DNA methylation level of CpG island (left panel) and CpG shore (right panel) regions between proteomic subtypes. Height of the column represents the mean value of DNA methylation level of all samples in the corresponding proteomic subtype, and the error bar represents the 95% confidence interval. *P*-values are calculated with Student’s *t*-test. **h** Representative enriched pathways based on genes overlapping with CNA gain (red) or loss (blue) regions in each proteomic subtype. **i** Dot plots visualizing representative enriched KEGG pathways based on cognate genes of differentially methylated sites in each proteomic subtype. **j** Bar plots comparing the proportion of patients with TNM staging (left panel) and BcR/BcPD (right panel) between proteomic subtypes. *P*-values are calculated with Pearson’s *χ*^2^ test. **k** Kaplan–Meier survival curves for SRFS of the three proteomic subtypes. See also Supplementary Fig. S4 and Tables S5–S7.

To provide insight into molecular commonalities and heterogeneities of MTC tumors, we then sought to derive a subtype classification of Chinese MTCs based on global proteomic data. Among the 102 MTC tumors, unsupervised clustering identified three proteomic clusters with distinct molecular features (Materials and Methods; Fig. 3a; Supplementary Table S5).

To be specific, Cluster-I (32.4% of all tumors) was characterized by enrichment of multiple pathways relevant to cellular metabolism, and was thereby named as the Metabolic subtype (Fig. 3b). Cluster-II, constituting 35.3% of tumors, showed elevated protein level of basal factors, such as CD44, KRT5, KRT6B, KRT6C, and KRT14. Moreover, protein-specific elevation of eukaryotic translation initiation (EIF) complex and cytoskeleton members indicated higher translational activity in this subtype (Fig. 3b, c). Taken together, Cluster-II was classified as the Basal subtype. On the other hand, Cluster-III (32.4% of all tumors) was named as the Mesenchymal subtype, as it was featured by predominant upregulation of extracellular matrix (ECM)-associated proteins and pathways (Fig. 3b, d).

The proteomic subtypes displayed differences in genetic and epigenetic patterns. (i) *RET*^M918T^-driven tumors were enriched in the Mesenchymal subtype, especially when compared with the Metabolic subtype (42.4% vs 18.2%, *P* = 0.030, Fisher’s exact test). Conversely, *RAS* mutations occurred more frequently in the Metabolic subtype (Metabolic vs Mesenchymal, 27.3% vs 3.0%, *P* = 0.013, Fisher’s exact test) (Fig. 3e). (ii) With regard to somatic copy number alteration (SCNA), the Metabolic subtype revealed the highest level of genome instability, while on the other hand, the Basal subtype had a relatively stable genome with significantly fewer copy number changes than other clusters (Fig. 3f; Supplementary Table S6). (iii) At the epigenomic level, the Mesenchymal subgroup might reveal a relatively higher level of DNA methylation (Fig. 3g). In addition, pathway enrichment analyses revealed concordant regulation trends at the SCNA, methylation and phosphoproteomic levels (i.e., Focal Adhesion in the Mesenchymal subtype; Fig. 3h, i; Supplementary Fig. S4). These results above indicated that mutations, copy number changes and epigenetic modifications may together participate in the determination of MTC phenotypes.

The proteomic subtypes also differed in clinicopathologic and prognostic characteristics. Compared with the Basal subtype, the Mesenchymal subtype revealed a more advanced TNM staging (*P* = 0.005), increased likelihood of BcR/BcPD (*P* = 0.035), and a compromised SRFS (*P* = 0.005), possibly representing a group of more aggressive tumors (Fig. 3j, k; Supplementary Table S7). By contrast, the Metabolic subtype appeared to show an intermediated degree of malignancy across the three subtypes.

Taken together, these discrepancies across the proteomic subtypes prompt us to further drill down into their respective multi-omics profiles that may reveal the tumor heterogeneity of MTC at a deeper level (details of each subtype are summarized in Table 1).

**Table 1:** Highlights of clinical relevance and proteogenomic hallmarks for MTC subtypes.

Subtype	Metabolic	Basal	Mesenchymal
Heredity	Hereditary MTC: 30.3%	Hereditary MTC: 30.6%	Hereditary MTC: 12.1%
Prognostic relevance	Intermediate TNM staging and prognosis between the other two subtypes	Earlier TNM staging; Better prognosis	Heavier burden of lymph node metastasis; More advanced TNM staging; Poorer prognosis
Driver mutation	*RET* (all variants, 60.6%) Higher prevalence of *RAS* mutation	*RET* (all variants, 83.3%) Intermediate prevalence of *RAS* or *RET*^M918T^ mutation	*RET* (all variants, 81.8%) Higher prevalence of *RET*^M918T^ mutation
SCNA	Highest level of chromosomal instability; Enrichment of CNV losses/deletions in DDR genes	Relatively stable genome	Median
Global DNA methylation level	Relatively lower	Median	Relatively higher
Proteogenomic signature	Activated MAPK and PI3K/Akt/mTOR signaling pathways; Enhanced cell cycle signaling; Higher HRD score	Higher degree of neuroendocrine differentiation	Activated STAT3 signaling; Upregulated targets of multiple approved or investigational TKIs

### Activated oncogenic pathways, enhanced cell cycle signaling and HRD signature as hallmarks of the Metabolic subtype

**Fig. 4:**
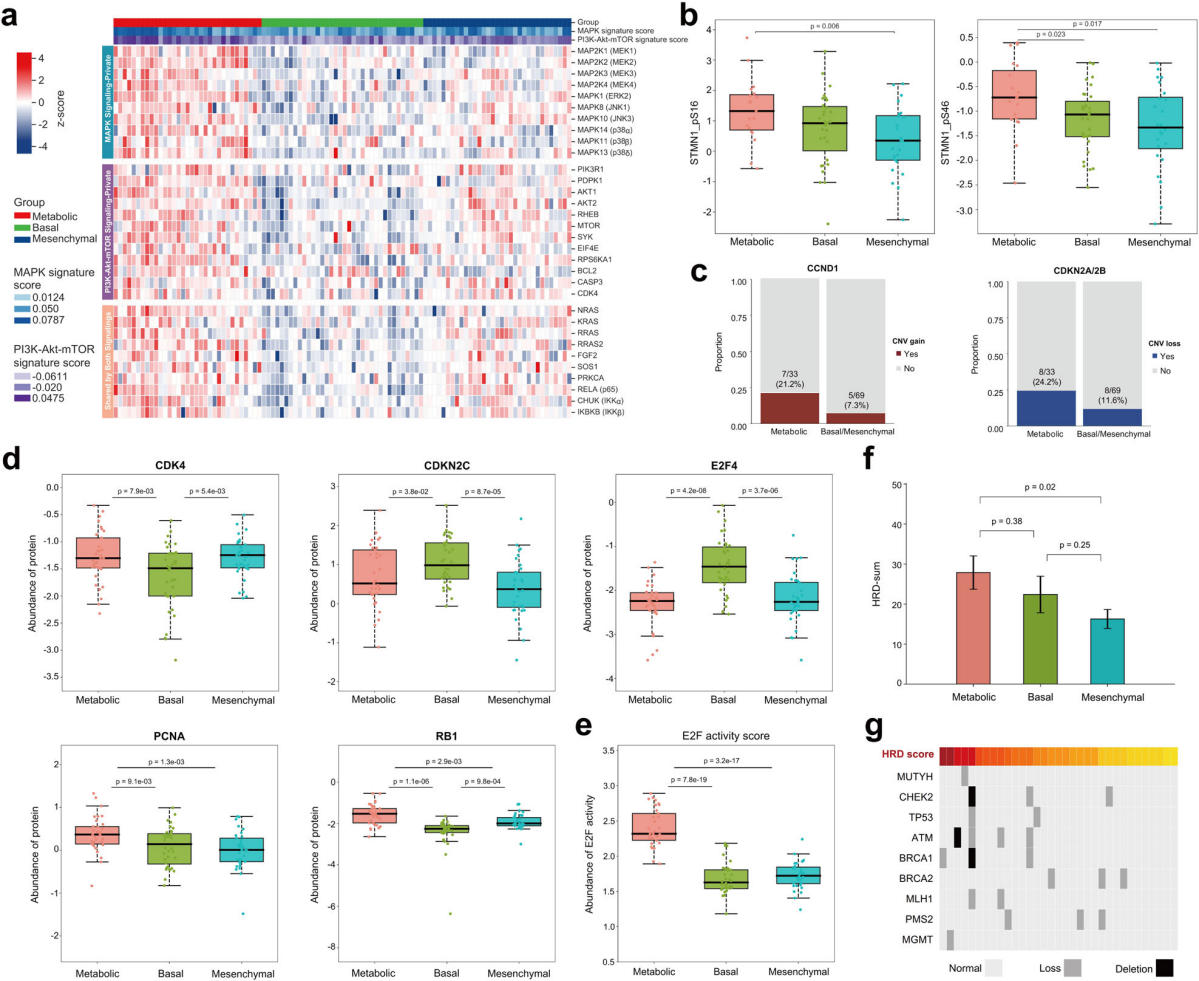
Proteogenomic insights into the Metabolic subtype reveal activated oncogenic pathways, enhanced cell cycle signaling and HRD signature. **a** Heatmap showing the abundance of key proteins in the MAPK pathway and/or PI3K/Akt/mTOR pathway. For each sample, ssGSEA enrichment score of MAPK- or PI3K-related pathways represents the sample’s own MAPK or PI3K/Akt/mTOR signature score, which is annotated above the heatmap with details shown on the left. **b** Boxplots showing the abundance of STMN1 S16 and S46 phosphosites across the three proteomic subtypes. For the boxplots: line in the box indicates the median; box borders correspond with the first and third quartiles (25th and 75th percentiles); whiskers extend 1.5 times the interquartile range; outlier data are shown as dots. **c** Distribution of CCND1 gains (left panel) and CDKN2A/CDKN2B losses (right panel) between the Metabolic subtype and other subtypes. **d** Boxplots showing the abundance of key cell cycle-related proteins CDK4, CDKN2C, E2F4, PCNA and RB1 across the three proteomic subtypes. *P*-values are calculated with Student’s *t*-test. For the boxplots: line in the box indicates the median; box borders correspond with the first and third quartiles (25th and 75th percentiles); whiskers extend 1.5 times the interquartile range; outlier data are shown as dots. **e** Boxplot comparing the E2F activity score across the three proteomic subtypes. *P*-values are calculated with Student’s *t*-test. For the boxplot: line in the box indicates the median; box borders correspond with the first and third quartiles (25th and 75th percentiles); whiskers extend 1.5 times the interquartile range; outlier data are shown as dots. **f** Histogram comparing the HRD score across the three proteomic subtypes. *P*-values are calculated with Student’s *t*-test. **g** Distribution of copy number losses/deletions of common DDR-related genes in the Metabolic subtype. The 33 Metabolic tumors are arranged in descending order based on HRD score. See also Supplementary Fig. S5 and Table S8.

We then dissected the multi-omics portraits of each subtype to explore their distinct biological hallmarks. Among the three clusters, single-sample gene set enrichment analysis (ssGSEA) based on global proteomic data revealed enhanced MAPK signaling activities in the Metabolic subtype (Supplementary Fig. S5). As shown in Fig. 4a, this subtype had increased level of multiple proteins not only in the classical ERK cascade, but also in the alternative JNK/p38 cascade. Meanwhile, we also observed elevated phosphorylation levels at serine 16 and 46 sites on STMN1, which is an important member of the MAPK pathway (Fig. 4b). In addition, tumors in this subgroup also exhibited a higher PI3K-Akt-mTOR activity with elevated abundance of proteins belonging to this pathway or those involved in both PI3K/Akt/mTOR and MAPK signaling pathways (Fig. 4a). Notably, prior studies have demonstrated the value of mTOR and/or RAF/MEK inhibition in certain MTC cell lines or patients^26–29^. Altogether, these results suggested that stronger activation of MAPK and PI3K/Akt/mTOR pathways may be an important biological feature of Metabolic tumors, which warrants further validation for their therapeutic values.

In addition, activated cell cycle signaling might be another hallmark of this subtype. With regard to genomic aberrations, *CCND1* gains (encoding cyclin D1) were noted in 21.2% of Metabolic tumors versus in only 7.3% of other subtypes (*P* = 0.041), while a higher frequency of *CDKN2A*/*CDKN2B* losses (encoding cyclin-dependent kinase inhibitor 2A/2B) was confirmed (Metabolic vs others, 24.2% vs 11.6%) (Fig. 4c). At the proteomic level, upregulation of cell cycle positive regulators CDK4, PCNA and RB1, and downregulation of negative regulators CDKN2C and E2F4 were observed, respectively (Fig. 4d). Consistent with these two independent omics, a significantly increased E2F activity score, inferred from the RNA-Seq data, also strongly indicated cell cycle activation in this subtype (Fig. 4e).

Furthermore, more careful investigation of this subtype indicated a higher Homologous Recombination Deficiency (HRD) score (range: 0–85) (Fig. 4f), representing increased genomic instability that accords with the results in Fig. 3f. Despite a lack of *BRCA1*/*BRCA2* pathogenic mutations, copy number losses/deletions of common DNA damage repair (DDR)-related genes (*CHEK2*, *MUTYH*, *TP53*, *ATM*, *BRCA1*, *MLH1*, *MGMT*) were observed in the vast majority of HRD-high (HRD score ≥ 42, 5/6, 83.3%) patients in this subtype (Fig. 4g; Supplementary Table S8).

### Proteogenomic insight of the Basal subtype indicates a subset of tumors retaining a higher degree of neuroendocrine properties

**Fig. 5:**
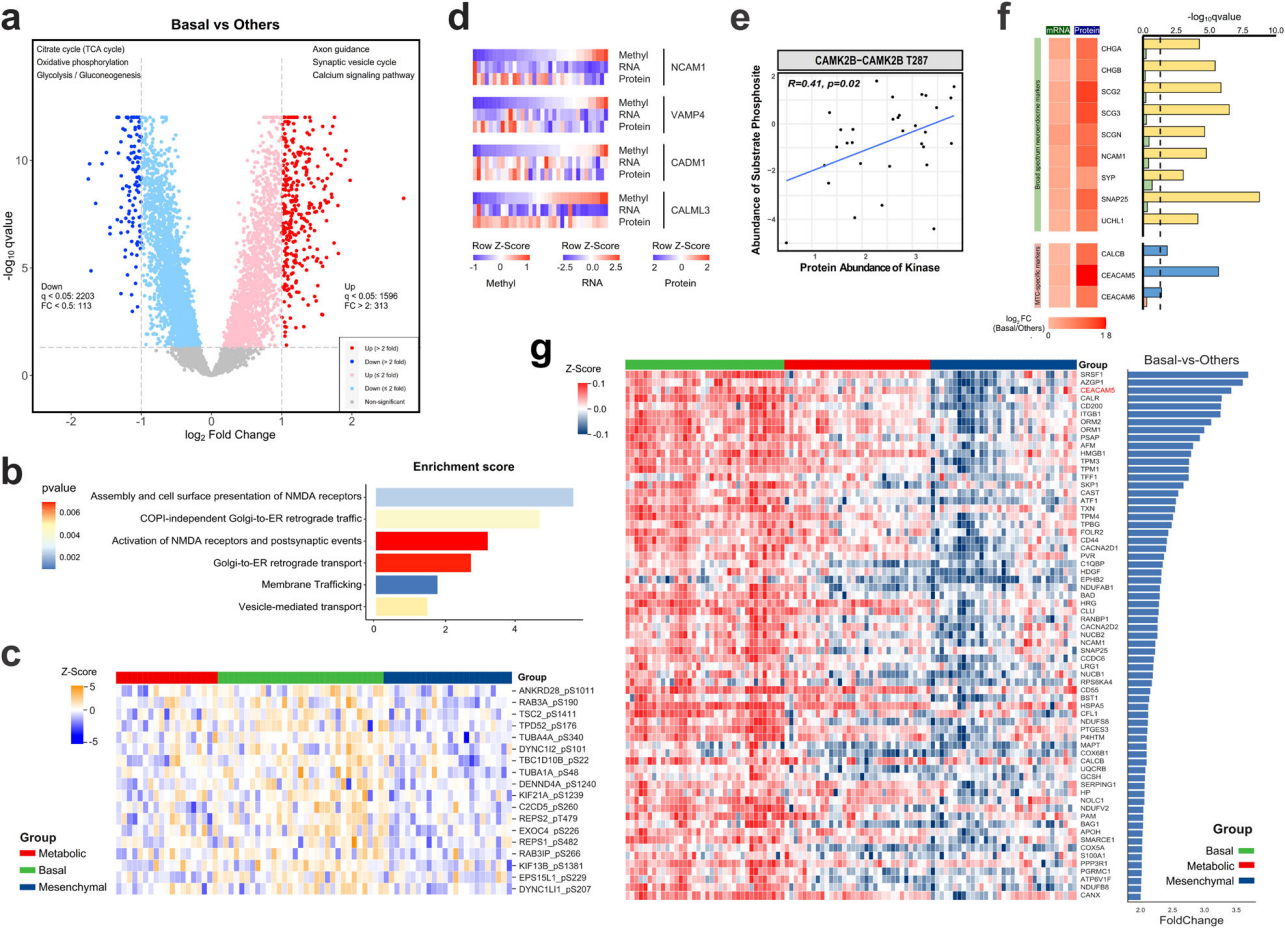
Proteogenomic insights into the Basal subtype reveal enhanced neuroendocrine properties. **a** Volcano plot showing differences in protein abundance between the Basal subtype and other subtypes (Student’s *t*-test). Representative KEGG pathways for > 2-fold upregulated and downregulated proteins are listed. **b** Bar plots showing representative enriched Reactome pathways using 138 significantly upregulated phosphoproteins in the Basal subtype. **c** Heatmap showing the abundance of phosphosites involved in the Reactome pathway Vesicle-mediated transport across the three proteomic subtypes. **d** Heatmaps showing the *cis*-effect of DNA methylation on cognate mRNA and protein abundance in four neurotransmitter conduction-related genes (*NCAM1*, *VAMP4*, *CADM1*, *CALML3*) in Basal tumors. Methylation level (β-value), mRNA abundance (FPKM) and protein abundances were displayed in this heatmap after normalization by row Z-score. **e** Scatter plot showing the correlation between the abundance of CAMK2B protein and CAMK2B T287 phosphosite. Pearson’s R and *P*-values are shown within the plot. **f** Differential mRNA and protein abundance of MTC-specific biomarkers and broad-spectrum neuroendocrine markers between the Basal subtype and other subtypes. *P*-values are calculated by Student’s *t*-test for global proteomic data, or calculated by DESeq2 based on the negative binomial distribution for RNA-Seq data. The right panel shows the –log_10_-transformed *P*-values of the two omics (upper bar for proteomic data, lower bar for RNA-Seq data). **g** Heatmap showing the abundance of 67 candidate drug target proteins of the Basal subtype. See also Supplementary Table S9.

Next, we focused on biological characteristics of the Basal subtype by leveraging multiplatform data, in which a clearly enhanced neuroendocrine-related molecular signature was discovered. For global proteins, 1596 (24%) were significantly increased and 2203 (33%) were significantly decreased in Basal tumors (*q* < 0.05). The 313 proteins increased by > 2-fold than other subtypes were enriched in synaptic transmission processes such as axon guidance, synaptic vesicle cycle and calcium signaling pathway, while in line with the indolent biology of this subtype, the 113 decreased proteins (> 2-fold) were concentrated in cellular energy metabolism including oxidative phosphorylation, citrate cycle and glycolysis (Fig. 5a; Supplementary Table S9). Likewise, for phosphoproteomic profiles, we observed a significant upregulation of pathways and phosphoproteins related to transport and exocytosis process of secretory proteins, such as Golgi-to-ER retrograde transport, membrane trafficking and vesicle-mediated transport in Basal tumors (Fig. 5b, c), further strengthening its intensified secretion function.

Aligned with the neuroendocrine signature, further investigation revealed two important trans-omic regulations exclusive in Basal tumors. First, a negative *cis*-effect of CpG island methylation on both mRNA and protein abundance was found in four genes related to neurotransmitter conduction (*NCAM1*, *VAMP4*, *CADM1*, *CALML3*) (Fig. 5d). Second, the inferred kinase–substrate network highlighted a positive association between the abundance of CAMK2B protein (Ca^2+^/calmodulin-dependent protein kinase II beta) and its own phosphosite at Thr287 (Pearson’s R = 0.41; *P* = 0.02, Fig. 5e), indicating a potential role of CAMKII autophosphorylation, a commonly recognized Ca^2+^-independent activation manner to crosslink postsynaptic proteins^30,31^, in tumor development of Basal MTCs.

Upon further searching the molecular profiles across these three clusters, we found that almost all major neuroendocrine tumor biomarkers detectable in our study were upregulated in the Basal subtype at both mRNA and protein levels, particularly at the protein level (Fig. 5f). Notably, this increase not only included common MTC-related biomarkers carcinoembryonic antigen (CEA, also called CEACAM5), calcitonin-related polypeptide beta (CALCB) and CEA cell adhesion molecule 6 (CEACAM6), but was also seen in other broad-spectrum neuroendocrine markers such as chromogranin A/B (CHGA/CHGB), synaptophysin (SYP), secretogranin II/III (SCG2/3) and neural cell adhesion molecule 1 (NCAM1, also called CD56) (Fig. 5f). These findings actually reveal a remarkably stronger neuroendocrine capability per unit volume of Basal tumors. Combined with its favorable prognosis, we suggest that the Basal subtype may represent a group of tumors retaining a higher degree of neuroendocrine differentiation, or in other words, more resembling normal C cells.

To explore potential drug targets for this subtype, we then investigated the characteristic upregulated proteins of Basal tumors (fold change (FC) > 2, *q* < 0.05 vs other subtypes), and searched these characteristic proteins (*n* = 67) in the DrugBank database (Materials and Methods). We found that CEA was the only protein among them meeting the following criteria, (i) strongly upregulated in Basal tumors (FC > 3, ranked third among the 67 proteins; Fig. 5g) and (ii) had clinically accessible drugs (approved or investigational, not just experimental), so it might be a candidate therapeutic target for Basal tumors, but it still warrants further validation.

### Candidate biomarkers and enriched targets of tyrosine kinase inhibitors in the Mesenchymal subtype

**Fig. 6:**
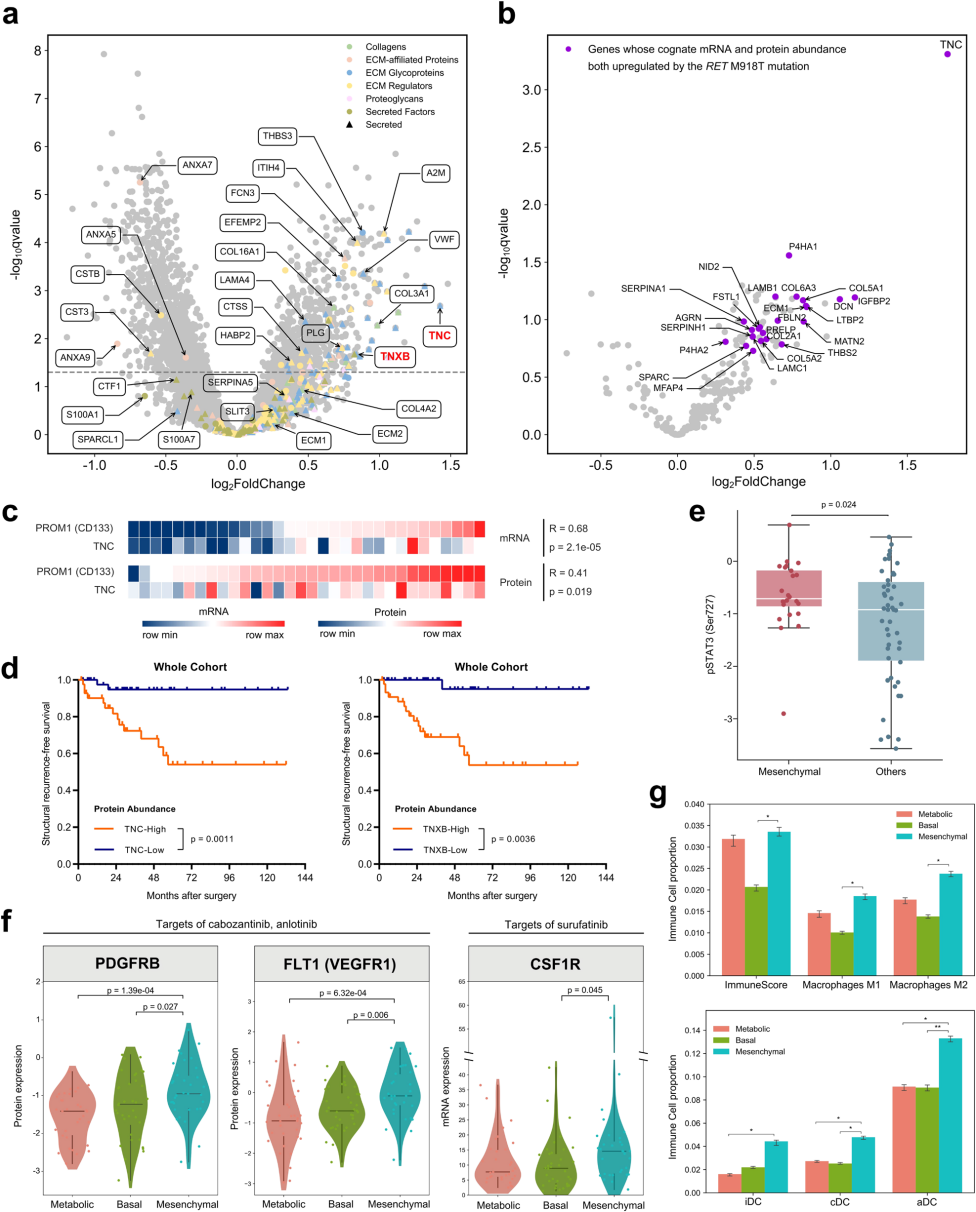
Proteogenomic insights into the Mesenchymal subtype reveal candidate biomarkers and enriched targets of TKIs. **a** Volcano plot showing differential protein abundance between the Mesenchymal subtype and other subtypes. Matrisome proteins and subclassifications are annotated, and two tenascin family members TNC and TNXB with large fold change are highlighted in red color. **b** Volcano plot showing differential abundance of matrisome proteins between *RET*^M918T^-mutated tumors and other tumors. Dots highlighted in purple color indicate genes whose cognate mRNA and protein abundances are both upregulated in *RET*^M918T^ tumors. **c** Heatmaps showing Pearson’s correlation between mRNA or protein levels of TNC and PROM1 (CD133) in the Mesenchymal subtype. **d** Kaplan–Meier survival curves comparing SRFS between TNC-high and TNC-low tumors (left panel), or between TNXB-high and TNXB-low tumors (right panel) in the whole cohort. Median abundances of TNC and TNXB are used as the cutoff value for high and low expression. *P*-values are calculated by log-rank test. **e** Boxplot showing differential abundance of phosphorylated STAT3 at SER727 between the Mesenchymal subtype and other subtypes. *P*-values are calculated with Student’s *t*-test. For the boxplot: line in the box indicates the median; box borders correspond with the first and third quartiles (25th and 75th percentiles); whiskers extend 1.5 times the interquartile range; outlier data are shown as dots. **f** Violin plots showing the protein abundances of PDGFRB, FLT1 (VEGFR1), and mRNA abundance of *CSF1R* across the three proteomic subtypes. *P*-values are calculated by Student’s *t*-test for proteomic data, or calculated by DESeq2 based on the negative binomial distribution for RNA-Seq data. **g** Histograms showing ImmuneScore and infiltration of M1-, M2-phenotype macrophages (upper panel), immature dendritic cells (iDCs), conventional dendritic cells (cDCs), activated dendritic cells (aDCs) (lower panel) as estimated by in silico xCell devolution. *P*-values are calculated with Student’s *t*-test, and are annotated as follows: ***P < 0.001, **P < 0.01, *P < 0.05. See also Supplementary Fig. S6 and Tables S10, S11.

The relatively unfavorable outcome of the Mesenchymal subtype motivated us to shed light on its diagnostic biomarkers and treatment targets. As illustrated in Fig. 6a and Supplementary Table S10, over one quarter of matrisome proteins (68/263, 25.9%)^32^ were significantly upregulated in the Mesenchymal subtype, the majority (56/68, 82.4%) of which were secretable proteins that may serve as putative salivary or blood biomarkers^33^.

Among these secretable proteins, we noticed that two tenascin family members tenascin-C (TNC) and tenascin-X (TNXB) showed prominent enrichment in Mesenchymal tumors, ranking 2nd and 60th across all 6607 quantifiable proteins in fold increase (Fig. 6a; Supplementary Fig. S6a, b). Meanwhile, we performed immunohistochemistry (IHC) staining of TNC using previously established tissue microarrays (TMAs)^34,35^. Among the 27 patients in this TMA overlapping with the present study cohort, the proportions of TNC positivity in Mesenchymal, Basal, and Metabolic subtypes were 66.7% (6/9), 14.3% (1/7), and 27.3% (3/11), respectively (representative IHC images of each subtype are shown in Supplementary Fig. S6c). In particular, in our proteomic data, TNC was confirmed to be the most strongly upregulated matrisome protein in *RET*^M918T^-driven tumors, while it also appeared to be positively correlated with the abundance of a classical tumor stemness marker PROM1 (CD133) in Mesenchymal tumors (Fig. 6b, c). These findings above prompted us to further focus on their prognostic value, where we found that higher TNC or TNXB expression (median as cutoff) was not only associated with advanced staging (TNC: *P* = 1.36e–05; TNXB: *P* = 4.28e–04), but also predicted a remarkably poorer SRFS (TNC: *P* = 0.0011; TNXB: *P* = 0.0036) in the whole study cohort (Fig. 6d; Supplementary Fig. S6d). Therefore, we suggest that TNC or TNXB may have the potential to be candidate biomarkers and prognostic indicators for MTC tumors, although larger independent cohorts are needed for further validation.

Consistent with increased tumor stemness and mesenchymal formation in Mesenchymal tumors^36^, we observed enhanced STAT3 signaling (represented by increased p-STAT3 level vs other subtypes, *P* = 0.024) in this subtype (Fig. 6e)^37^, which is a critical transcription activator in angiogenesis. Consistently, key angiogenic factors PDGFRB and VEGFR1 (FLT1) were also significantly upregulated in Mesenchymal tumors (Fig. 6f), which are not only drug targets for the FDA-approved tyrosine kinase inhibitor (TKI) cabozantinib, but also the targets of several other TKIs in clinical trials for advanced MTC such as sorafenib, lenvatinib, sunitinib, etc. (Supplementary Fig. S6e). In addition, these two tyrosine kinase receptors are also targets for anlotinib, a TKI approved for the treatment of advanced MTC in mainland China^38^ (Fig. 6f; Supplementary Fig. S6e).

Furthermore, surufatinib has shown promising efficacy in patients with locally advanced or metastatic MTC and other neuroendocrine tumors in previous clinical trials^39–41^. In addition to VEGFR1, surufatinib also targets colony stimulating factor 1 receptor (CSF1R) that mediates the polarization of tumor-associated macrophages (TAM, M2 phenotype). Inhibition of CSF1R could re-polarize M2 macrophages into the anti-tumorigenic M1 phenotype, which has been identified as a key anti-tumor mechanism of this drug^42^. Notably, despite a lack of quantifiable proteomic level of CSF1R (due to < 50% samples had available data of this protein in mass spectrometry), our transcriptomic profiling revealed a significantly upregulated *CSF1R* mRNA level in Mesenchymal tumors, as well as enhanced infiltration of M2-, M1-phenotype macrophages, dendritic cells (DCs) and increased ImmunoScore inferred by in silico xCell deconvolution^43^ (Fig. 6f, g; Supplementary Table S11).

## Discussion

Molecular underpinnings of medullary thyroid carcinoma have not been comprehensively characterized owing to the rarity of this neuroendocrine malignancy. Through collective and long-term efforts, our cooperative group OTTA has established a large cohort of fresh-frozen samples of MTC. Utilizing this platform, we herein present a multicenter multi-omics study incorporating five platforms (genomics, transcriptomics, epigenomics, proteomics and phosphoproteomics) that provides novel insights into the biological understanding of MTC, and also contributes a rich resource of sequencing data to fulfill the needs of future translational research.

Previous genomic analyses have greatly broadened our knowledge of molecular events relevant to this cancer^25,44,45^. However, these studies are based on either small patient cohorts or targeted region sequencing instead of WES, leading to a difficulty in detecting rare mutations. In our study, independent of the well-recognized dominant driver genes *RET* and *RAS*, we expanded genetic drivers of MTC by identifying uncommon *BRAF* and *NF1* alterations, whose downstream transcriptomic and proteomic consequences suggest potential oncogenic roles in this disease. Notably, the relationship between the *BRAF* proto-oncogene and MTC pathogenesis has been scarcely described, with only two case reports detecting *BRAF*^V600E^ mutation in *RET*-negative MTC tumors, and one study reporting a potentially activating *BRAF*–*PARP12* gene fusion^46–48^. However, despite an absence of the well-characterized V600E mutation, *BRAF* mutations in our MTC cohort occurred in two non-hotspot sites G469A and T599dup, suggesting novel drivers in this cancer.

Although rare, co-occurrence of neurofibromatosis type I and MTC or its precancerous condition C-cell hyperplasia has been documented in previous literatures, several of which have identified *NF1* with or without *RET* germline mutations by peripheral blood DNA sequencing^49–54^. However, none of these studies used MTC tumor samples for genetic testing, resulting in a lack of direct evidence for the underlying cause of MTC in this syndrome. In our study cohort, we also reported a typical neurofibromatosis type I patient with concomitant MTC. Unlike previous studies, after ruling out other potential genetic drivers, we detected a somatic de novo truncating mutation in the *NF1* gene directly in his MTC tumor, and further confirmed the downstream outcome of this mutation at transcriptomic and proteomic levels. Collectively, our data reveals the first direct evidence for *NF1*-driven MTC tumorigenesis.

In the past decade, targeted therapies for advanced MTC have achieved a stepwise progression from a barren land to a non-selective paradigm and then to a more precise management based on the status of *RET* mutation. Objective response rate (ORR) observed with TKIs in advanced MTC patients is high, being ~80% with selective RET inhibitors and ~50% with anti-angiogenic TKIs accompanied by an extended response duration. However, the rate of complete response (CR) is still far from satisfactory (~5% for both selpercatinib and pralsetinib)^6,7^. In addition, even for selective RET inhibitors that have just started to be used clinically in recent years, primary and secondary resistance in tumors is emerging and receiving increasing attention^9,55,56^. It is widely acknowledged that resistance to TKIs is universal, therefore emergence of resistance to RET inhibitors is anticipated in all treated patients sooner or later^56^. Meanwhile, a tumor’s driver mutation is not solely responsible for its biological behavior. For a given driver such as *RET*^M918T^, this hazardous mutation has been reported to involve in multiple signaling pathways including Wnt, NFκB, Notch, JAK/STAT, and MAPK pathways^57^, while epigenetic modifications, post-transcriptional or post-translational regulations may all contribute to the differences in downstream proteomic profiles. In combination with the facts above, identifying drug targets, especially at the protein level, may be promising. In the present study, beyond the genetic level, our data suggest that the proteomic heterogeneity existing across MTC tumors may provide potential therapeutic vulnerabilities.

Among our proteomic clusters, PI3K/Akt/mTOR and MAPK pathways exhibited boosted activities in the Metabolic subtype, both have been implicated as major cascades that contribute to MTC/MEN2 pathogenesis partially through dysregulated cellular metabolism^58–61^. This uncovers the mechanism why this phenotype is metabolically active, and also attracts us to discuss the potential therapeutic value of these two important oncogenic pathways. Notably, mTOR inhibitors have been widely used in the treatment of neuroendocrine tumors (NETs), for example, everolimus has been approved in pancreatic, gastrointestinal and lung NETs^62^. Although its application in MTC is still in the exploratory stage, previous trials have suggested that a subpopulation of advanced MTCs can indeed benefit from everolimus administration^27,29,63^. Nevertheless, more data are warranted to support whether these beneficiaries belong to Metabolic tumors. In addition, enhanced cell cycle activity and increased HRD score were also identified as the hallmarks of the Metabolic subtype. *RB1* and *CDKN2A* have been associated with responses to cell cycle inhibitors^64^, while a higher HRD score has been correlated with responses to DNA damaging agents, such as PARP inhibitors^65^. Therefore, whether these hallmarks could provide therapeutic opportunity also deserves further validation.

The Basal subtype was characterized by a greater retention of neuroendocrine properties. MTC carcinogenesis is partially caused by de-differentiation from functional C cells to tumor cells^66,67^, thus it is reasonable to assume that Basal tumors are at an earlier step of this process and retain a higher degree of differentiation, which helps explain why patients with Basal tumors have a better prognosis. Based on this feature, we focused our attention on neuroendocrine markers as potential treatment targets, where CEA that preferentially overexpressed in Basal tumors was finally selected. Anti-CEA radioimmunotherapy has been reported to bring responses or even survival improvement in a proportion of metastatic MTCs^68,69^, while antibody-drug conjugates (ADCs), the state-of-the-art antibody therapy, is starting to be applied across a wide range of CEA-expressing solid tumors^70,71^. However, their real therapeutic efficacy in MTCs still needs further experiments or clinical trials for validation.

Hallmarks of the Mesenchymal subtype, including a slight global DNA hypermethylation, enrichment of the hazardous *RET*^M918T^ mutation, and STAT3 signaling activation, may be causally related and collectively serve as molecular determinants of poorer prognosis in Mesenchymal tumors. Meanwhile, we found that stromal secretable proteins TNC and TNXB, in addition to their potential diagnostic value, also revealed strong prognostic significance, implying their downstream regulatory roles underlying the aggressive behaviors of this phenotype. Consistently, our data were consolidated by previous findings showing tenascin family as a pan-cancer stromal biomarker and negative prognosticator that promotes cell proliferation, metastasis, angiogenesis, and epithelial–mesenchymal transition^72,73^. In terms of treatment targets, to our surprise, key target proteins for multiple TKIs, including cabozantinib, vandetanib, anlotinib and surufatinib, were preferentially upregulated in Mesenchymal tumors. Although these drugs are either approved or already under investigation in advanced MTCs, they tended to yield only poor to moderate efficacy in unselected patients (Supplementary Fig. S6e), suggesting a demand for more accurate selection of potential beneficiaries. Since several TKIs have already been approved for MTC, investigators can retrospectively or prospectively evaluate the effects of these drugs in MTC patients with Mesenchymal tumors.

Although the tumor heterogeneity and different biological signatures among MTC proteomic subtypes suggest potential treatment possibilities, further evidence for validation must be added before we translate our hypotheses into clinical trials, and these constitute several limitations of our study that we must acknowledge. First, future studies should be planned to investigate clinically accessible biomarkers (i.e., by IHC or blood assay) to discriminate proteomic subtypes. Second, our hypothesis and conclusions were mainly based on in silico analyses. There are very few commercially available MTC cell lines at present, and the few cell lines (e.g., TT or MZ-CRC-1) represent MTCs driven by specific point mutation. If we would like to mimic different proteotypes or validate drug targets proposed in our study, or even identify novel drug targets, multiple primary cell lines, patient-derived xenograft or organoids are still warranted. However, these tumor models may have not been successfully constructed in MTC as no relevant literature was published yet. Thus, these technologies were not applied in this study. Third, this study was performed on primary tumor samples and extended disease may appear or may need to be treated some years or decades later. Biological behavior and treatment response between extended disease and primary tumor may be different. Fourth, kinetic parameters (such as serum calcitonin or CEA doubling time) are useful predictive markers of MTC tumor burden and are confirmed to correlate with the probability of disease relapse. However, different assays among different hospitals, or even at different periods in the same hospital caused a strong diversity of reference values and results, making it difficult to apply in this multicenter study including MTC samples spanning over a ten-year period. Fifth, the present work is mostly global proteome-centric. In-depth excavation of other omics, especially for RNA-Seq, methylome, and phosphoproteome, is still warranted to provide more profound insights into the molecular basis of MTC. Sixth, an independent validation cohort of MTC samples, although very difficult for this rare malignancy, may help further enhance the strength of evidence of our conclusions.

In conclusion, our current work broadens our biological understanding of MTC and brings forth therapeutic hypotheses that may underpin future preclinical studies and clinical trials toward molecularly guided therapy of this rare cancer. Moreover, this study provides the largest collection of comprehensively profiled MTC tumors to date, and could serve as an important resource for further investigation of MTC biology and therapeutic vulnerabilities.

## Materials and methods

### Patient selection and sample acquisition

We initially enrolled 109 MTC patients undergoing primary curative surgery from 2007 to 2020 in five Chinese tertiary hospitals in the OTTA consortium, including Fudan University Shanghai Cancer Center (FUSCC, *n* = 85), Zhejiang Cancer Hospital (ZJCH, *n* = 21), Changzhou Second People’s hospital (CZSPH, *n* = 1), Taizhou People’s hospital (TZPH, *n* = 1), and Peking University Shenzhen Hospital (PUSH, *n* = 1). After a careful pathological review, seven patients were excluded according to the following reasons: (A) unavailable paired normal thyroid tissue or blood (*n* = 1); (B) questionable MTC pathology (*n* = 3); (C) extremely low tumor cellularity (*n* = 2); (D) tumor-contaminated normal thyroid tissue (*n* = 1). Finally, 102 MTC patients were included in the study cohort (Supplementary Fig. S1).

As for sample acquisition, tumor samples or normal thyroid tissue (at least 2 cm away from the tumor margin) were snap-frozen in liquid nitrogen within 30 min of surgical resection, and then kept at –80 °C at the Tissue Bank of each hospital until further processing. Peripheral blood samples were collected the day prior to surgery and were stored at –80 °C until use. The middle section of all tumor samples and normal thyroid tissues were resected and processed for hematoxylin and eosin (H&E) staining. Under the examination of two independent pathologists, tumor samples and normal thyroid tissues were carefully checked for cellularity and tumor contaminants, respectively. After quality control, tumor samples from a single site of primary lesion were cryopulverized and aliquoted for genomics and proteomics. Notably, comparison between primary MTC tumor and normal thyroid tissue does not reflect the cancer initiation process because MTC-derived C cells are sparsely distributed in the normal thyroid gland, which instead consists predominantly of follicular epithelial cells. Therefore, normal thyroid samples were not used in other sequencing platforms except as germline control in WES, just like peripheral blood samples.

All patients were re-staged per the 8th edition of American Joint Committee on Cancer staging system. BcR or BcPD was defined as recurrent or persistent elevation of serum calcitonin or CEA, respectively. In a similar manner, SR or SPD was defined as recurrent or persistent histological/radiographic evidence of disease after surgery, respectively. Accordingly, SRFS was defined as the time from initial surgery to first occurrence of structural disease. In survival analyses, patients without SR were censored from the time of last follow-up.

The study methodologies conformed to the standards set by the Declaration of Helsinki and were approved by the Institutional Review Board of Fudan University Shanghai Cancer Center (License# 050432-4-1911D). Written informed consent was provided from each patient in the study cohort.

### WES data analysis

#### Exome sequencing alignment and quality assessment

The raw data from 102 paired tumor/non-tumor samples were demultiplexed and then converted into FASTQ format. Sequencing reads data were checked for quality and adaptor/primer sequence contamination first by FastQC (version 0.11.5). Before filtering, the proportion of sequenced bases with ≥ Q20 value ranged from 97.07% to 98.78% (average: 98.13%), while GC-content ranged from 47.88% to 55.69% (average: 51.32%). The raw reads were then filtered by Trimmomatic (version 0.36) to remove low-quality reads in the following order: (1) remove adaptor sequences; (2) reads with N (non-AGCT) ≥ 5; (3) remove bases with an average quality < 20 in a 4-base sliding window; (4) After trimming, reads with a length < 75 bp or with an average quality < 15 were dropped out. After these quality control processes, an average of 171.73 M clean reads and 98.78 M clean reads were obtained for tumor samples and NT/blood samples, respectively.

Clean reads were then aligned to the GRCh37/hg19 human reference genome with Burrows–Wheeler Aligner (BWA; version 0.7.17, http://bio-bwa.sourceforge.net)^74^, and were also annotated with information from GRCh38/hg38.p12 to ensure the data consistency in multi-omics integrative analyses. The resulting BAM files were preprocessed using version 201711 of Sentieon tools (https://www.sentieon.com/). PCR duplicates were discarded by Picard (version 2.25.0, http://broadinstitute.github.io/picard/). Due to the systematic technical errors from sequencing machines, base quality scores of reads were then recalibrated by the BaseRecalibrator tool from GATK (version 4.2.0.0, https://software.broadinstitute.org/gatk/)^75^.

On the whole, our WES profiling achieved a mean on target sequencing coverage of 178× for tumor samples and 112× for NT/blood samples, consistent with the recommendations for WES^76,77^.

#### Calling of putative somatic driver mutations

Somatic mutations, including single nucleotide variants (SNVs) and insertions and deletions (INDELs), were detected by MuTect2 (embedded in GATK, version 4.2.0.0) with the default parameters. Then these mutations were further annotated using ANNOVAR (version 2019 Oct 24, http://annovar.openbioinformatics.org/en/latest/)^78^ and COSMIC (version 85, https://cancer.sanger.ac.uk/cosmic/)^79^. Germline variants were filtered from database of the 1000 Genomes^80^, NHLBI Exome Sequencing Project (ESP6500), Exome Aggregation Consortium (EXAC), and Genome Aggregation Database (gnomAD). The filtered mutations (including SNVs and INDELs) were further used to identify significantly mutated genes (SMGs) by MuSiC (version 0.4.1–1, https://gmt.genome.wustl.edu/packages/genome-music/) with default parameters^81^, where SMGs referred to genes with a significantly higher mutation rate than the background mutation rate. Notably, there is a long-held view and increasing evidence supporting that most neuroendocrine tumors (including MTC) distinctively arise from a single driver event, either inherited or acquired^82^. Therefore, these SMGs were subjected to a further filtering process where we strived to filter out passenger mutations or likely artifacts with a strict criteria, as listed below.

In brief, our criteria could be summarized into a three-step approach. First, we retained somatic mutation calls that (1) were seen in reads in both directions; (2) quality score ≥ 20; (3) variant sequencing coverage ≥ 10×; (4) variant supporting reads ≥ 5 in the tumor sample; (5) variant allele frequency (VAF) ≥ 0.05; (6) minor allele frequency (MAF) < 0.01 in either East Asian or all population of known population databases including Genome Aggregation Database (gnomAD), NHLBI Exome Sequencing Project (ESP6500) and 1000 Genomes Project^80^; (7) nonsynonymous variants or variants in splice region, including missense mutation, nonsense mutation, nonstop mutation, splice site or translation start site, frameshift or in-frame INDELs. Second, apart from the putative drivers identified in previous steps, variants not in the Catalogue Of Somatic Mutations In Cancer (COSMIC) database were excluded. Third, consistent with a TCGA study of neuroendocrine tumors (pheochromocytoma and paraganglioma)^20^, all these filtered calls were manually reviewed one by one in previous literature, and only known hotspot mutations that have been reported as definite driver mutations in MTC or other cancers were retained as putative drivers.

#### Calling of germline RET driver mutations

Germline *RET* mutations were detected from germline reference normal samples (normal thyroid tissue or blood) by HaplotypeCaller (embedded in GATK, version 4.2.0.0), and were then annotated with ANNOVAR (version 2019 Oct 24). As described above, the first step of germline *RET* mutation calling process was similar to that of somatic mutations. Next, to screen out hereditary MTCs and remove likely artifacts, all these filtered calls were manually reviewed and were retained only when reported as the cause of MEN2 in previous literatures.

#### Mutual exclusivity analysis of somatic RET and RAS mutations

Fisher’s exact test was used to detect mutual exclusivity of *RET* and *RAS* somatic mutations.

#### Exome-based SCNA analysis

To assess SCNA, WES data from tumor and paired normal samples were analyzed by Control-FREEC (version 8.0)^83^ to obtain segmented copy number calls and allelic imbalances. Then these segmented calls were applied as the input of Genomic Identification of Significant Targets in Cancer (GISTIC) version 2.0.23 to identify significant focal SCNA events^84^. Consistent with a previous study^85^, the GISTIC2.0 program was run with the following settings (-smallmem 1; -broad 1; -conf 0.95; -cap 3.5; -armpeel 1; -savegene 1; -gcm extreme -ta 0.2; -td 0.2; -rx 0; -genegistic 1; -brlen 0.7) to estimate log_2_-transformed, gene-level or segment-level SCNA ratios, where the GISTIC calls comprised -2 (deletion), -1 (loss), 0 (diploid), 1 (gain), and 2 (amplification). We then identified subtype-specific SCNA cytobands of each proteomic subtype, defined as cytobands that had a significantly higher frequency of SCNA events in one subtype than the other subtypes (Fisher’s exact test, *P* < 0.05). In addition, we annotated those subtype-specific cytobands that having at least one gene with *cis*-effect on its cognate protein with Pearson’s *R* > 0.4, *P* < 0.05 between a gene’s SCNA ratios and protein abundance.

#### Evaluation of HRD score

We used the scarHRD R package to determine the levels of HRD based on three genomic scar signatures: number of telomeric allelic imbalances (NtAI), loss of heterozygosity (LOH) and large-scale transitions (LST)^86,87^. Briefly, NtAI refers to allelic imbalance extending to the subtelomeric region > 11 Mb in size but not crossing the centromere. LOH is defined as either deletion of one allele (copy loss LOH) or deletion and simultaneous duplication of the remaining allele (copy neutral LOH), resulting in the loss of one of the two alleles at a heterozygous locus. The size of affected segments with LOH is generally longer than 15 Mb but shorter than a whole chromosome. LST was defined as the number of break points between regions exceeding 10 Mb after filtering out regions shorter than 3 Mb^88^.

### RNA-Seq data analysis

#### RNA-Seq data quality control and generation with FPKM

The raw data from 101 tumor samples were then demultiplexed and converted into FASTQ format. Sequencing reads were checked for quality and adaptor/primer sequence contamination first by FastQC (version 0.11.5). Before filtering, the proportion of sequenced bases with ≥ Q30 value ranged from 90.66% to 96.03% (average: 93.63%), while GC-content ranged from 45.22% to 62.33% (average: 49.42%). The raw reads were then filtered by Trimmomatic (version 0.36) to remove low-quality reads in the following order: (1) remove adaptor sequences; (2) remove bases with an average quality < 3 at the 5’ or 3’ ends; (3) remove bases with an average quality < 15 in a 4-base sliding window from the 5’ end; (4) after trimming, reads with length < 50 bp were dropped out. After these steps, an average of 80.69 M clean reads for each sample were retained for subsequent analyses. Subsequently, clean reads of RNA-Seq data were aligned to the reference genome GRCh38/hg38.p12 (patch release 12) using HISAT2 (version 2.1.0)^89^, and the count number of each gene was obtained by HTSeq-count (version 0.11.2)^90^. Then the Fragments Per Kilobase of exon model per Million mapped fragments (FPKM) value was calculated using the TopHat-Cufflinks pipeline based on the count number^91^. The differences in mRNA abundance were calculated by DESeq2. If multiple testing was performed, *P*-values were adjusted using the Benjamini–Hochberg procedure.

After quality control, RNA-Seq resulted in an average of 80.76 M clean reads per sample. RNA-seq data analysis identified a total of 19,598 protein-coding genes with an average of 16,742 genes per sample. The distribution of gene expression was largely consistent across the 101 samples subjected to RNA-Seq analysis, suggesting a high degree of stability of our sequencing platform and homogeneity in the transcriptomic profiling of MTC tumors (Supplementary Fig. S2e).

#### RNA-Seq variant calling

For RNA-Seq variant calling, all reads that passed the quality control (QC) were first aligned using BWA (version 0.7.17, http://bio-bwa.sourceforge.net). SAMtools (version 1.9, http://www.htslib.org) was used to sort the above results and prepare the aligned bam files. Afterwards, variant calling was conducted with BCFtools (version 1.9, http://www.htslib.org) and all obtained variants were annotated with snpEff (version 3.4p, http://pcingola.github.io/SnpEff/). During the whole process, highly accurate variants were filtered with parameters: DP > 10, QUAL > 40, and MQ (RMS Mapping Quality) > 20.

#### Prediction of tumor purity

Tumor purity was predicted with the ESTIMATE package^12^ in RStudio using FPKM values of the normalized RNA-Seq data.

#### Evaluation of E2F activity score

According to a previous study^15^, E2F hallmark gene sets were queried from the MSigDB Hallmark gene sets^92^. For these genes, ssGSEA was conducted for tumor samples using FPKM value of RNA-Seq data via the GSVA R package^93^. Prior to data normalization, read counts of 0 were treated as NAs. Enrichment scores were calculated from ssGSEA and were regarded as the E2F pathway activity score.

### DNA methylation data analysis

#### EPIC methylation array data quality control

The raw IDAT files were processed by GenomeStudio Methylation Module software (version 1.8, Illumina, San Diego, CA). Consisting of 865,918 probes, the raw data were subjected to quality control process using the R package ChAMP (version 2.12.4, https://bioconductor.org/packages/release/bioc/html/ChAMP.html)^94^ in the following order: (1) we removed probes with detection *P* > 0.01; (2) removed probes with a bead count < 3 in at least 5% of samples; (3) removed non-CG probes; (4) removed probes containing an SNP; (5) removed probes aligning to multiple locations (MultiHit probes); (6) removed probes located on either the X or Y chromosome. After these steps, the clean data were generated with a total of 697,795 probes, and were then normalized by BMIQ in the ChAMP package (version 2.12.4).

After normalization, the data were used to calculate DNA methylation levels, displayed as *β*-values ranging from 0 to 1, corresponding to unmethylated and methylated sites, respectively. CpG sites were mapped to the human reference genome (GRCh37/hg19) using the annotation file provided by the manufacturer (R package “IlluminaHumanMethylationEPICanno.ilm10b2.hg19”), and further curated and translated to GRCh38/hg38.p12 (patch release 12)^95^.

#### Comparison of global DNA methylation levels between the proteomic subtypes

Histograms were used to show the difference in global DNA methylation level in CpG island or CpG shore regions across proteomic subtypes. For each sample, the mean value of all *β*-values in either CpG island or CpG shore regions was calculated to represent the global DNA methylation level of this sample, as displayed in the vertical coordinate. The height of the column represented the mean value of the DNA methylation level of all samples in this proteomic subtype, and the error bar represented the 95% confidence interval, reflecting the dispersion of the global DNA methylation level of different samples in each subtype. The difference of global DNA methylation level was evaluated using Student’s *t*-test, and a *P* < 0.05 was considered to be statistically significant.

#### Pathway enrichment analysis based on DNA methylation profiling of the three proteomic subtypes

Differentially methylated sites (DMS) (*β* > 0.1, adjusted *P* < 0.05) were identified in each subtype respectively in comparison to all other samples (one subtype vs other two subtypes). The KEGG pathway enrichment analysis was performed on the cognate genes of these DMSs for each subtype using a hypergeometric test.

### Proteome and phosphoproteome data analysis

#### Quantification of global proteome and phosphoproteome data

Proteins or phosphopeptides with missing values in < 50% of the total samples were regarded as quantifiable and were subjected to further data normalization. Specifically, we identified 6607 quantifiable proteins and 3073 quantifiable phosphopeptides that were detected in more than half of the samples subjected to global proteome and phosphoproteome, respectively (≥ 51 out of 102 samples for global proteome; ≥ 37 out of 74 samples for phosphoproteome). The XIC value of each protein or phosphopeptide was normalized with the column-median centering method to correct for equal loading across samples, and were then log_2_-transformed. Subsequently, K-nearest neighbor (KNN) algorithm was applied for missing value imputation.

The very long and continuous operating time of the mass spectrometer (12 consecutive days for global proteomics and phosphoproteomics, respectively) makes it difficult to guarantee absolute stability of instrument condition. During the data checking, we observed that 11 samples (FUSCC-03, FUSCC-11, FUSCC-12, FUSCC-14, FUSCC-15, FUSCC-17, FUSCC-18, FUSCC-19, FUSCC-21, FUSCC-23, FUSCC-32) clustered together and significantly deviated from the other samples in the PCA plot (data not shown). These 11 samples happened to be tested continuously in sequential order on LC-MS/MS. For this reason, we retrospectively checked the total ion chromatogram of each sample, and observed intermittent instabilities in the instrument when these 11 samples were being loaded and tested. Therefore, the resulting batch effects from the 11 samples were corrected by ComBat function in SVA R package with default parameters^96^.

#### Unsupervised clustering for global proteomic data

Based on all 6607 quantifiable proteins from the global proteomic data, ssGSEA was performed using the Kyoto Encyclopedia of Genes and Genomes (KEGG) dataset to assess the Enrichment Score (ES) of all signaling pathways for each sample. Next, unsupervised hierarchical clustering was performed according to the average Euclidean distance metric of ES for each sample, which classified the 102 tumor samples into three initial clusters with distinct patterns of enriched signaling pathways.

To further reduce the “noise” of subtyping and to highlight the heterogeneity between subtypes, we aimed to further distinguish the functional differences of these initial clusters. Therefore, we then defined the characteristic signaling module of each cluster, by selecting pathways with the ratio of mean ES values between a given cluster/other clusters > 1.2 from the enriched signaling pathways mentioned above. The three characteristic signaling modules generated on this basis contained a total of 1485 proteins, among which we finally selected the top 60% proteins (*n* = 891) according to the coefficient of variation (CV) across samples for downstream analyses (Supplementary Table S5). Based on the average Bray–Curtis dissimilarities among the expression abundance of these 891 proteins, unsupervised hierarchical clustering was conducted to identify proteo-typical clusters with the following parameters: pivot_kws = None, method = “average”, metric = “braycurtis”. The heatmap was plotted by the clustermap() function of seaborn Python package 0.21.2. In summary, the 102 MTC tumor samples were divided into three subgroups with sample numbers of 33, 36, and 33, respectively.

#### Estimation of BRAF score based on global proteomic data

In this study, we used a *BRAF* score to quantify the extent to which the downstream signaling of a given sample resembles the *BRAF*^V600E^-mutant profiles^21^. The *BRAF* score, based on the expression profiles of 58 genes, is a modified version of a 71-gene-based *BRAF*^V600E^-*RAS* scoring algorithm derived from a TCGA study of papillary thyroid cancer^97^. Of the 58 *BRAF* score-related genes, a total of 25 genes (*QSOX1*, *ASAP2*, *PPL*, *STK17B*, *ETHE1*, *FN1*, *ANXA1*, *PROS1*, *SFTPB*, *PTPRE*, *ITGA3*, *SDC4*, *GBP2*, *AHR*, *PDLIM4*, *CTSC*, *BID*, *CDC42EP1*, *LLGL1*, *SEL1L3*, *ANKLE2*, *SYT12*, *PRICKLE1*, *TMEM43*, *PLEKHA6*) were quantifiable in our global proteomic data, whose protein abundances were then used for score calculation with AddModuleScore function of the Seurat R package (v3.1.4) following the methods described in a previous study^21^.

#### Evaluation of MAPK and PI3K-Akt-mTOR signaling activity

The ssGSEA analysis was carried out based on the global proteomic data of each tumor sample. Query gene sets for ssGSEA included all pathways from KEGG datasets. In ssGSEA, the matrix of protein abundances for a given sample is rank-normalized, and an ES is then generated using the Empirical Cumulative Distribution Functions (ECDF). Finally, the ES of each gene set was normalized to account for the size of that gene set, yielding a normalized enrichment score (NES). Activities of MAPK signaling and PI3K-Akt-mTOR signaling were evaluated based on their respective NES.

#### Pathway enrichment analysis between Basal tumors and all other tumors

Differential expression analysis for global proteomic data was carried out between Basal tumors and other tumors using Student’s *t*-test. *P*-values were adjusted by the Benjamini–Hochberg procedure and features were considered significant with an adjusted *P*-value (FDR, also called *q*-value) < 0.05. Finally, in the Basal group, we identified a total of 313 proteins upregulated by at least two-fold, and 113 proteins downregulated by at least two-fold, respectively, whose cognate genes were separately aligned to KEGG pathway enrichment analysis using the hypergeometric test by “Phyper” function available in the stats R package. Pathways with a *P*-value < 0.05 were regarded to be significantly regulated. For the KEGG enrichment analysis of upregulated and downregulated proteins, we listed three characteristic pathways each.

One-way ANOVA was applied to identify phosphopeptides with significantly different abundance (*P* < 0.05) among the three proteomic subtypes, which were then compared between Basal tumors and other tumors. These phosphopeptides with FC > 1.2 in the Basal group were further selected, whose cognate genes were included to derive enriched pathways using the Reactome databases. *P*-values were calculated by the R function “Phyper” function based on the hypergeometric test.

#### Identification of potential drug targets for the Basal subtype

First, we attempted to identify characteristic proteins of the Basal subtype. The differences of protein abundance between Basal tumors and other tumors were compared by Student’s *t*-test with *P*-values adjusted by Benjamini–Hochberg procedure. Proteins with FC > 2 and *q* < 0.05 were considered significantly upregulated and regarded as characteristic proteins of this subtype. Second, these proteins were then searched in the DrugBank database (https://go.drugbank.com/) to see if they had been already identified as drug targets^98^. As a result, a total of 67 candidate drug target proteins were retrieved. Third, as the drugs recorded in DrugBank were often clinically unavailable or only experimental, we manually reviewed the therapeutic targets recorded in the DrugBank database for each of these proteins, in order to identify therapeutic targets with truly clinically accessible drugs (approved or investigational in solid tumors, not merely experimental). Finally, CEA having both a high upregulation (FC = 3.42) and investigational drug (anti-CEA antibody-drug conjugate, e.g., labetuzumab) was the only candidate that met this criteria.

### Multi-omics data analysis

#### mRNA–protein correlation

The gene-wise mRNA–protein correlation for all genes quantifiable in both omics (*n* = 6454) was computed using Spearman’s correlation with statistical significance set at FDR < 0.05. On the other hand, the sample-wise mRNA–protein correlation was calculated as the median of gene-wise correlation of all genes with cognate mRNA–protein pairs in each sample.

Subsequently, using the cognate genes of significantly positively (Spearman’s *r* > 0, FDR < 0.05) or negatively (Spearman’s *r* < 0, FDR < 0.05) correlated gene-wise mRNA–protein pairs, KEGG pathway enrichment analysis was performed by R function “Phyper” based on the hypergeometric test. Signed –log_10_*P*-value was used as the ranking metric to identify KEGG pathways enriched for genes with low and high protein–RNA correlations, respectively, and 35 signaling pathways with the lowest *P*-values (highest –log_10_*P*-values) were selected to generate the ridge plot. In the ridge plot, the *x*-axis displayed the distribution of Spearman’s correlation coefficients of mRNA–protein pairs whose cognate genes were included in the 35 KEGG pathways (one per row). These pathways were listed in descending order of the median of the Spearman’s coefficients from top to bottom.

#### Comparison between global proteomic and transcriptomic data

We used information of all 4274 mammalian protein complexes recorded in the CORUM database (version 3.0, released in 2018) to make the comparison between global proteomic and transcriptomic data. For all 6607 quantifiable proteins in global proteome data, we fist calculated the expression correlations between each pair of proteins (a total of 21,822,921 protein–protein pairs). For all 19,645 quantifiable genes in RNA-Seq data, we also calculated the expression correlations between each pair of RNAs (a total of 54,162,592 RNA–RNA pairs). Correlations of each protein–protein pair and RNA–RNA pair were matched with the information recorded in the CORUM database.

The match results were considered to be true-positive if the predicted correlations existed in the CORUM database, otherwise they were regarded as false-positive. Similarly, true-negatives and false-negative results could be determined. Afterwards, a receiver operating characteristic (ROC) curve was plotted to compare the superiority of proteome and transcriptome in predicting CORUM protein complexes by “sklearn” function of Python version 0.21.2. In this analysis, two thirds of our samples were used as the training cohort, while the remaining one third of samples were used for validation.

#### Phosphosite-to-protein co-variation analysis

For the 74 MTC tumors with both proteome and phosphoproteome data, Pearson’s correlation coefficients were computed between phosphosite and cognate protein abundances in the three proteomic subtypes, respectively. Those phosphosite-to-protein pairs with Pearson’s coefficient > 0.2, and *P* < 0.05 were considered significantly co-regulated in each subtype. In total, 980 significantly co-regulated pairs were identified in the three proteomic subtypes, where 267, 557 and 501 pairs were identified in the Metabolic, Basal and Mesenchymal subtypes, respectively. Unsupervised hierarchical clustering with complete linkage was then performed using the Pearson’s correlation coefficient of each pair, and the 980 pairs could be classed into three major clusters. GO enrichment analysis was conducted using the cognate genes of these pairs, and the enriched terms/pathways (one specific cluster vs other clusters) were identified with a Benjamini–Hochberg FDR < 0.05, of which representative ones were annotated in the heatmap.

#### Survival analysis

Kaplan–Meier survival plots were used to describe SRFS of different genotypes or proteomic subtypes, and log-rank tests were used to evaluate the differences between survival curves. All these analyses were performed by GraphPad Prism version 8.4.0 (GraphPad Software).

### IHC staining and evaluation

#### Immunostaining experiment

IHC staining was performed to measure the level of TNC using the TMA slides that had been previously constructed, on which sections were cut from the representative tumor areas of > 200 MTC tumor samples^34,35^. In brief, the slides were subsequently deparaffinized and rehydrated, followed by quenching endogenous peroxidase activity and conducting heat-induced epitope retrieval. After preventing nonspecific antibody binding by using 5% normal goat serum, the slides were then incubated with TNC (1:200, ab108930, Abcam) at 4 °C overnight. A Dako REAL EnVision Detection System (horseradish peroxidase; HRP, Rabbit/Mouse, Agilent) was applied to stain the slides for 30 min at room temperature. The positive signal was finally detected after 3,3ʹ-diaminobenzidine coloration, hematoxylin counterstaining, and dehydration.

#### Immunostaining evaluation

The IHC-stained slides were screened by a KF-PRO-120 Digital Pathology Slide Scanner (Konfoong Biotech) and viewed with K-Viewer Digital Pathology System, version 1.5.3.1 (Konfoong Biotech). The immunostaining of each core was independently evaluated by two experienced pathologists (T.C. and Y.Z.) who were blinded to clinical parameters and outcomes. The cutoff for TNC positivity was decided according to proportion of staining in the whole tumor area (including mesenchyme), and was finally set as 5% based on the consensus of the two pathologists.

## Supplementary information

Supplementary Information

Supplementary Table S1

Supplementary Table S2

Supplementary Table S3

Supplementary Table S4

Supplementary Table S5

Supplementary Table S6

Supplementary Table S7

Supplementary Table S8

Supplementary Table S9

Supplementary Table S10

Supplementary Table S11

## Data Availability

For WES, RNA-Seq, and DNA methylation array, the raw data and processed datasets generated in this study are available at the Genome Sequence Archive (GSA) under accession ID PRJCA008783. For global proteomics and phosphoproteomics, the raw data and processed datasets generated in this study are available at the iProX via the accession number IPX0004234000.
