# Supplementary material for: Integrated proteogenomic characterization of medullary thyroid carcinoma
Source: Cell Discov. 2022 Nov 8;8:120. doi: 10.1038/s41421-022-00479-y (PMC9640541; doi:10.1038/s41421-022-00479-y)
Supplement: Supplementary file 1 — Supplementary Information [file 41421_2022_479_MOESM1_ESM.pdf]

Supplementary Materials and Methods

Genomic DNA and Total RNA Extraction

About 10~30 mg wet-weight for each tumor/NT sample or 200 μL for each blood sample was used for DNA extraction. According to the manufacturer's protocol, genomic DNA was extracted from tumor and normal thyroid tissues using the QIAamp 96 DNA QIAcube HT Kit (Qiagen), while peripheral blood DNA was isolated using TIANamp Genomic DNA Kit (Tiangen). DNA quantification was determined by the NanoDrop 2000 spectrophotometer (Thermo Scientific) and agarose gel electrophoresis, respectively.

About 10~15 mg wet-weight for each tumor sample was used for RNA extraction. Total RNA was extracted from cryopulverized tumor aliquots using the TRIzol reagent (Invitrogen). RNA purity and quantification were measured with a NanoDrop 2000 spectrophotometer (Thermo Scientific), and RNA integrity was evaluated by Agilent 2100 Bioanalyzer (Agilent Technologies). Samples with RNA integrity number > 6.0 were considered to be of high quality and acceptable for further sequencing.

Whole Exome Sequencing

For WES, qualified genomic DNA from the 102 tumor/non-tumor paired samples (tumor: n = 102; normal thyroid tissue: n = 86; blood: n = 16) were prepared for library construction (560 ng DNA for each sample). The WES libraries were then prepared and captured with an Agilent SureSelect Human All Exon v6 kit (Agilent Technologies) according to the manufacturer's protocol. Afterwards, pooled libraries were sequenced

on Illumina NovaSeq 6000 system (Illumina) with 150 bp paired-end reads.

RNA Sequencing

For RNA-Seq, 101 tumor samples in our study cohort had qualified total RNA for subsequent sequencing, and 500 ng RNA of each sample was used for library construction. Ribosomal RNA was depleted from total RNA with Ribo-Zero Gold rRNA Removal Kit (Illumina). Subsequently, the sequencing libraries were constructed with TruSeq Stranded Total RNA Library Prep Kit (Illumina) following the manufacturer's instructions, and library quality was assessed on an Agilent 2100 Bioanalyzer. Then the libraries were sequenced on an Illumina NovaSeq 6000 system with 150 bp paired-end read length.

Illumina Infinium MethylationEPIC BeadChip Array

After WES, a total of 78 tumor samples in our cohort had remaining tissue aliquots sufficient for DNA methylation array. About 10~25 mg wet-weight for each MTC tumor sample was used for DNA extraction, and 500 ng genomic DNA from each tumor was bisulfite treated using the EZ DNA methylation-Gold Kit (Zymo Research). Bisulfite-converted DNA was analyzed on an 8-sample version Infinium MethylationEPIC 850K BeadChip (Illumina) capturing > 850,000 methylation sites per sample. This EPIC array includes sample plating, bisulfite conversion, and methylation array processing. After scanning, the data were processed through an automated genotype calling pipeline provided by the manufacturer. The raw data were available

as IDAT files and a sample sheet.

Sample Preparation for Proteomic and Phosphoproteomic Analysis

Protein Extraction and Digestion

For all 102 tumors in our cohort, approximately 25-100 mg of each cryopulverized tumor sample was lysed with 300 μL SDS lysis buffer supplemented with 1mM PMSF, followed by 3 min of sonication (1 second on and 1 second off, 80 Watts power) (Ningbo Scientz Biotechnology). After sonication, the lysate was centrifuged at 15000 g, 4°C for 15 min twice to remove insoluble particles and precipitations, and protein concentration was determined by BCA assay (Thermo Scientific).

Based on the concentration determined by the BCA assay, 50 μg of protein per sample was used in subsequent process. The protein solution was incubated with 5mM dithiothreitol (DTT) at 55°C for 60 min, and was subsequently alkylated with a final concentration of 10 mM iodoacetamide for 30 min at room temperature (RT) in the dark. Followed by protein precipitation and collection, the precipitates were redissolved with 100 μL 100 mM triethyl ammonium bicarbonate (TEAB), and then digested by sequencing grade modified trypsin (Promega) at 1:50 enzyme-to-substrate ratio overnight at 37°C. The digested peptides were acidified by 100% formic acid (FA, Thermo Scientific) to 1% of the final concentration of FA to adjust the pH value to ~2. Finally, the tryptic peptides were desalted on reversed-phase C18-Reverse-Phase SPE Column (Waters) and dried by Speed-Vac (Thermo Scientific).

Phosphopeptide Enrichment by IMAC

A total of 74 tumor samples in our cohort had sufficient peptide material for phosphoproteome. Phosphopeptides were enriched from the digested peptide material (450 μg from each sample) using immobilized metal affinity chromatography (IMAC). The IMAC enrichment was performed with a High-Select™ Fe-NTA Phosphopeptide Enrichment Kit (Thermo Scientific) following the kit instructions. Briefly, 400 μg lyophilized peptides from 74 tumor samples with sufficient material were dissolved in 200 μL IMAC binding/wash buffer (80% acetonitrile [ACN], 0.1% trifluoroacetic acid [TFA]) and were then loaded onto an equilibrated spin column and mixed with the prepared resins. The peptide-resin mixture was incubated for 30 min by gently mixing every 10 min. Following centrifugation (1000 g, 30 s), the spin column was washed thrice using 200 μL binding/wash buffer and washed twice with 200 μL LC-MS grade water. Finally, the enriched phosphopeptides were eluted and dried by Speed-Vac.

High-pH RPLC Fractionation for Spectral Library

To prepare spectral libraries for MTC proteomic and phosphoproteomic analyses, an aliquot of unlabeled, trypsin-digested peptide material from individual tumor samples (102 for global proteomics, 74 for phosphoproteomics) was pooled and then subjected to reversed phase liquid chromatography (RPLC). Reverse phase separation was conducted on an Agilent Zorbax Extend RP column (150 mm × 2.1 mm column containing 5-μm particles) using the Agilent 1100 HPLC System. Mobile phases A (2% ACN in HPLC water, pH 10) and B (90% ACN in HPLC water, pH 10) were used for

RP gradient. The solvent gradient was set as follows: 2% B in 0-10 min; 2-5% B in 10-10.01 min; 5-20% B in 10.01-37 min; 20-40% B in 37-48 min; 40-90% B in 48-48.01 min; 90% B in 48.01-58 min; 90-2% B in 58-58.01 min; 2% B in 58.01-63 min.

Tryptic peptides were separated and collected into 40 fractions every minute within the period from 10 to 50 minutes. Next, these 40 fractions were concatenated into 10 fractions by combining 4 fractions that are 10 fractions apart (i.e., combining fractions #1, #11, #21, and #31; #2, #12, #22, and #32; and so on). For proteome spectral library, the post-concatenated 10 fractions were dried by Speed-Vac for further mass spectrometry (MS). For phosphoproteome spectral library, the 10 fractions were dried reconstituted in phopsho-loading buffer (2% TFA/60% ACN) for phosphopeptide enrichment as described above. Each fraction was analyzed individually with LC-MS/MS settings as described below.

LC-MS/MS Analysis

DDA-PASEF Mode to Generate Proteomic or Phosphoproteomic Spectral Library

We then generated proteomic and phosphoproteomic spectral library in data-dependent acquisition (DDA) mode. The benchmark and quality control process using the iRT Standard kit (Biognosys Inc.) were consistent with a previous study [1]. First, each post-concatenated fraction from RPLC (directly used for proteome, used after phosphopeptide enrichment for phosphoproteome) was spiked with iRT peptides and was then analyzed on an EASY-nLC 1200 nanoflow system (Thermo Scientific) with Aurora Series UHPLC emitter columns (75 µm × 250 mm) with nanoZero® +

CaptiveSpray Insert (IonOpticks). In brief, peptides were separated at a flow rate of 300 nL/min. Buffer A consisted of 0.1% FA in H_2O, and buffer B consisted of 0.1% FA in 80% ACN and 20% H_2O. For proteome, a 60-min gradient was set as follows, 0–45 min, 2-27% buffer B; 45–50 min, 27–46% B; 50–55 min, 46–100% B; 55–60 min, 100% B. For phosphoproteome, a 120-min gradient was set as follows, 0–90 min, 2-27% buffer B; 90–112 min, 27–46% B; 112–115 min, 46–100% B; 115–120 min, 100% B.

Next, proteomic and phosphoproteomic analyses were respectively performed on a hybrid trapped ion mobility spectrometry (TIMS) quadrupole time-of-flight (TOF) mass spectrometer (timsTOF Pro, Bruker Daltonics) via a CaptiveSpray nano-electrospray ion source. Using the parallel accumulation–serial fragmentation (PASEF) acquisition method, the timsTOF Pro system incorporates an extra dimension of ion mobility for peptide separation that provides significantly enhanced scan speed, specificity and sensitivity. The MS was operated in DDA mode for the ion mobility-enhanced spectral library generation with the following parameters. The peptides were eluted into MS with a fixed capillary voltage of 1.5 KV and 4.5 KV for proteomic and phosphoproteomic analyses, respectively. MS was recorded in the range from 100 to 1700 m/z with dry temperature of 180°C, and dry gas of 3L/min. The ion mobility was scanned from 0.85 to 1.3 Vs/cm2 for proteomic analysis and from 0.75 to 1.4 Vs/cm2 for phosphoproteomic analysis, while the collision energy was ramped linearly as a function of the mobility from 59 eV at $1/K_0 = 1.3$ Vs/cm^2 to 20 eV at $1/K_0 = 0.85$ Vs/cm^2 for both platforms.

Subsequently, the spectral library was generated based on the PulSar search engine

integrated into Spectronaut^{TM} (version 15.3.210906.50606, Biognosys Inc.) In brief, the DDA-PASEF data were searched against Human reference FASTA files downloaded from UniProt (released in May 2021, 20409 entries). The search parameters for global proteome were set as follows: Trypsyin/P enzyme with up to two missed cleavages, fixed modification of Carbamidomethyl (C), variable modification of Methionine oxidation, and 1% peptide spectrum match (PSM), peptide and protein level FDR for false identifications. For phosphoproteome, the search parameters were consistent with those in global proteome, except that phospho (STY) was also chosen as a modification. The phosphosite localization tool was then integrated into Spectronaut^{TM} to filter Class I (a localization probability cutoff > 0.75) phosphorylation sites which were regarded as highly reliable sites.

DIA-PASEF Mode to Get Proteomic and Phosphoproteomic Data

Tryptic peptide material (200 ng each) from 102 individual tumor samples, and IMAC-enriched phosphopeptide material (200 ng each) from 74 individual tumor samples were spiked with iRT peptides (Biognosys) and subjected to nano-LC MS/MS with basically similar parameters to that of DDA described above, except for minor changes made in the process of MS regarding ion mobility (0.6-1.6 Vs/cm2 for both global proteome and phosphoproteome) and dynamic collision energy (20-59 eV). (Supplementary Fig. S2a, b)

The DDA-PASEF spectral library was loaded onto Spectronaut^{TM} (version 15.3.210906.50606). Subsequently, 102 DIA runs for global proteomics and 74 DIA

runs for phosphoproteomics were analyzed by Spectronaut^{TM} to search the constructed DDA spectral library, respectively. In addition to the search parameters for the DDA library generation mentioned above, the main parameters of the software also included the following settings: retention time prediction type was dynamic iRT, interference on MS2 level correction was enabled, and cross run normalization was enabled. All results on protein and precursor levels were filtered based on a q-value cutoff of 0.01 (equivalent to FDR < 1%).

By searching the constructed library with Spectronaut^{TM}, a total of 64305 peptides from 7974 proteins were obtained for the proteomic analysis, 61179 of which were unique peptides. The phosphoproteomic analysis then yielded a total of 7417 phosphopeptides (covering 7566 confidently localized phosphosites), 7214 of which were unique phosphopeptides. The abundances of peptides were all quantified based on the area under the extracted ion chromatogram (XIC) peaks of all assigned fragments that passed filtering. For each protein with at least one matching unique peptide, the abundance was quantified using the Top-3 approach peptides abundance score. To be specific, the Top-3 approach calculated the total protein ion intensity for the respective proteins by using the median intensities from the three most intense peptides matching to a specific protein, if the number of unique peptides from a specific protein was < 3, then all the 1-2 peptides were used for calculation) [2-5]. For phosphoproteomic analysis, peptide grouping was performed based on modified peptides, and site-specific quantification was counted from the quantity of phosphorylation sequences according to Spectronaut^{TM} strategy (version 15.3.210906.50606). (Supplementary Fig. S2c, d)

Supplementary Figures

Figure S1

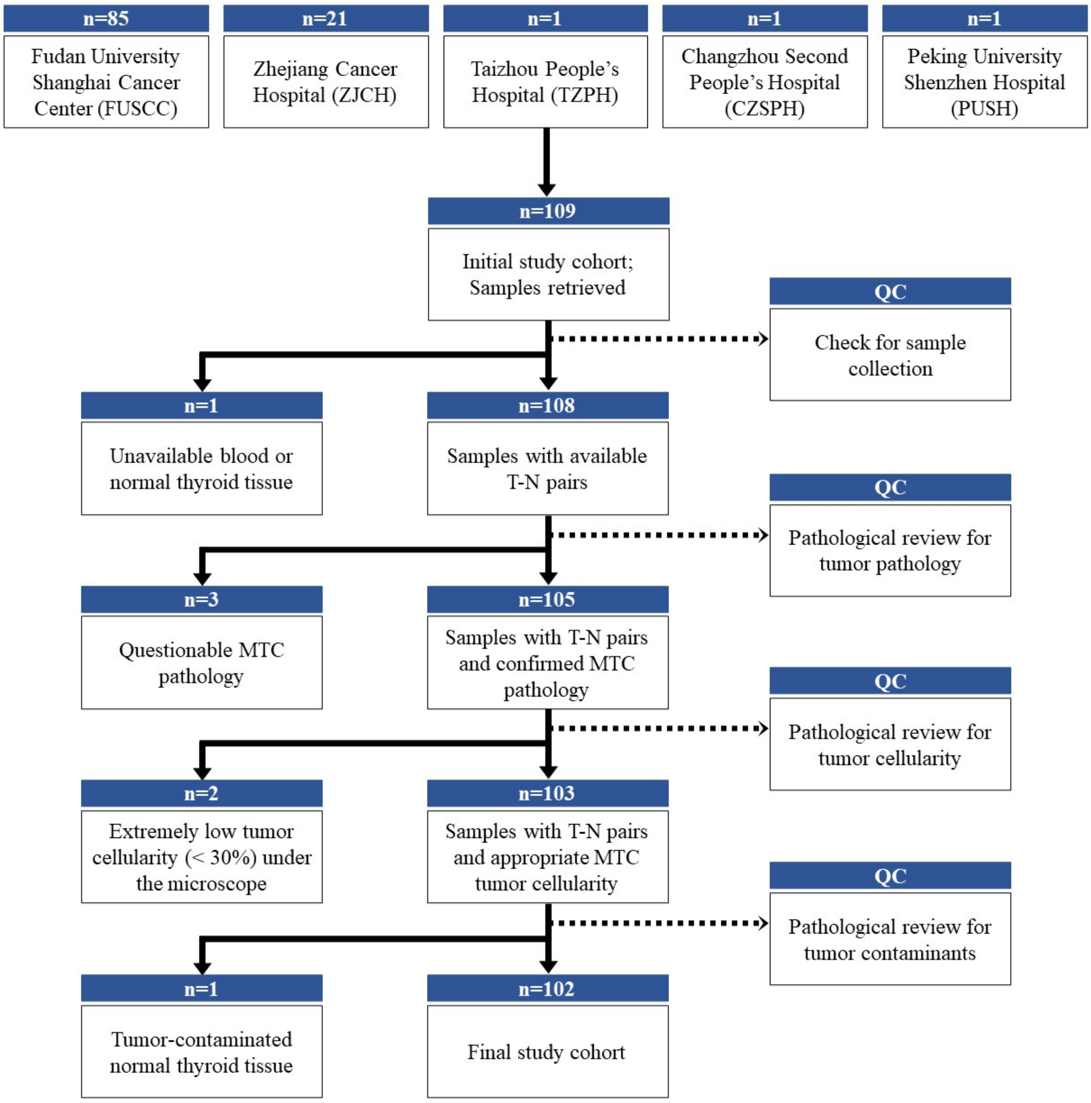

Supplementary Fig. S1 Flow Chart Showing the Selection Process of Our Study Cohort, Related to Methods.

Figure S2

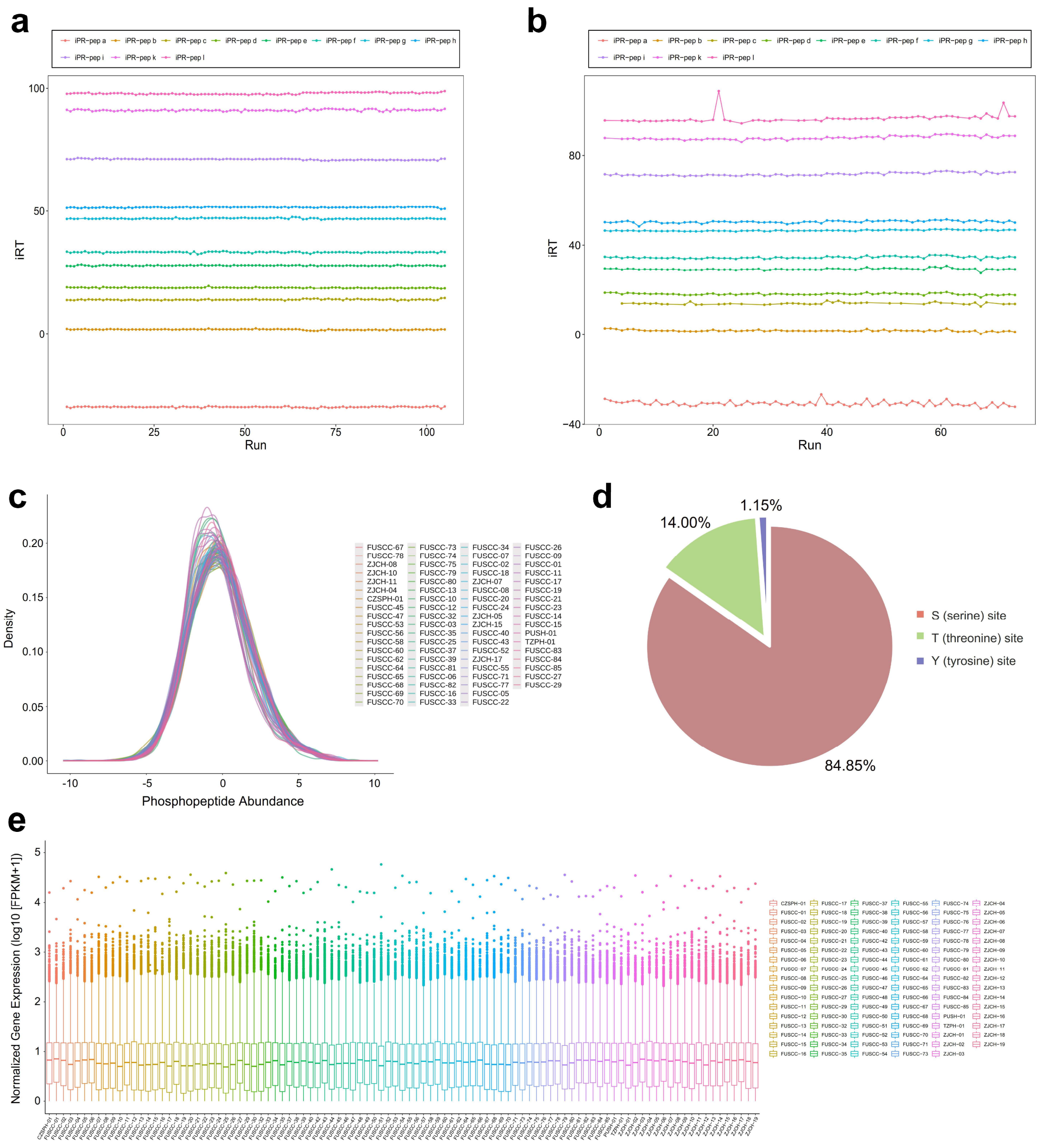

Supplementary Fig. S2 Quality Assessments for Global Proteome, Phosphoproteome and RNA-Seq Data, Related to Methods.

a, b Retention times of the 11 spiked-in peptides from the iRT kit (Biognosys) are plotted in the chronological order of data acquisition. Both plots show that the iRT runs are relatively stable in at least 10 of 11 spiked-in peptides for both (**a**) global proteome and (**b**) phosphoproteome. **c** Each curve in this density plot represents the probability distribution of the quantifiable phosphopeptide

abundance values of the sample included in phosphoproteome. **d** The sectors in the pie chart indicate the percentage of S (serine), T (threonine), and Y (tyrosine) modifications in the total phosphosites, respectively. **e** Boxplots show a high degree of stability of our sequencing platform and homogeneity in the transcriptomic profiling of MTC tumors in RNA-Seq data.

Figure S3

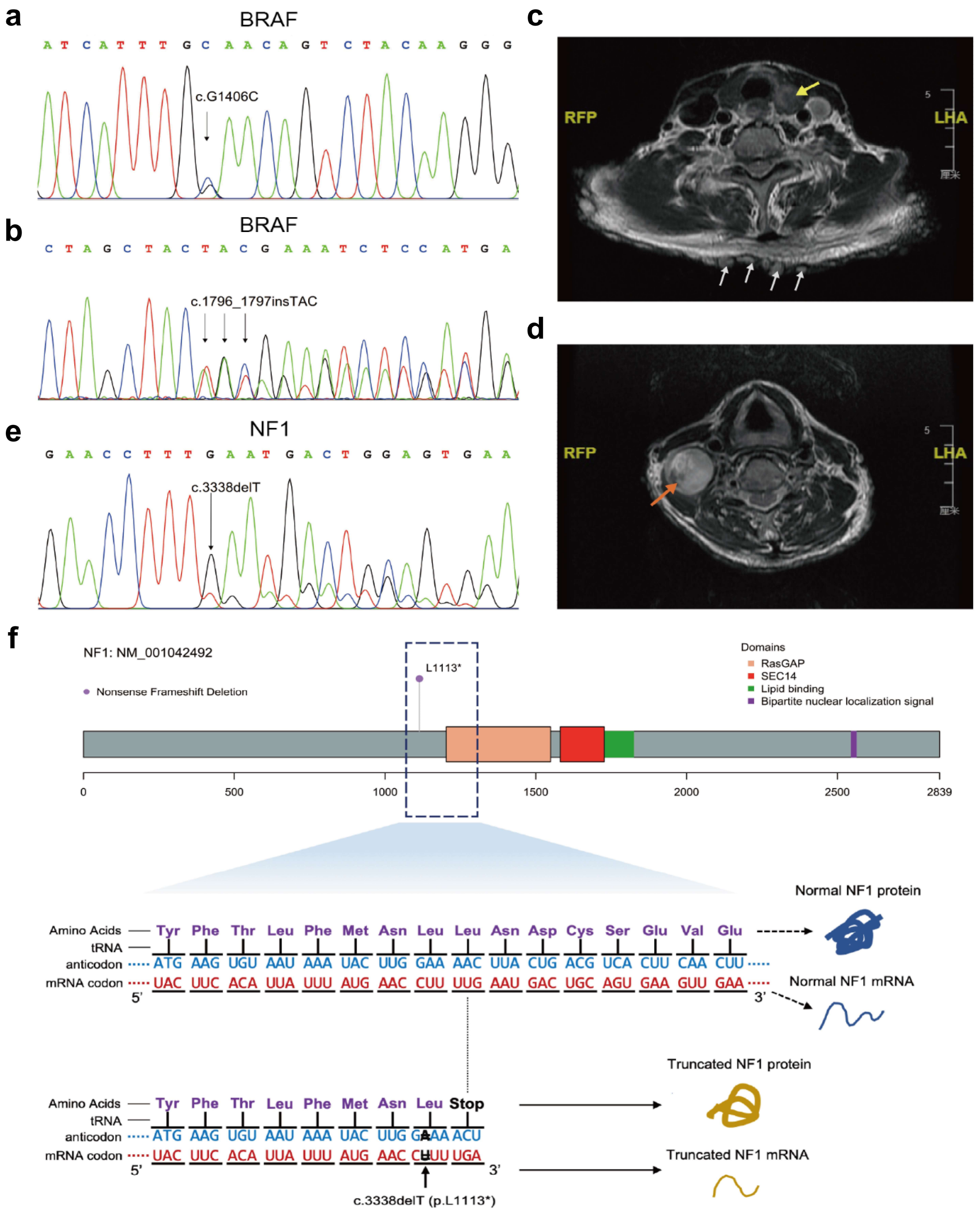

Supplementary Fig. S3 ***BRAF*** **and** ***NF1*** **Driver Mutations in the Study Cohort, Related to Fig. 2.**

a, b Sanger sequencing chromatograms showing somatic *BRAF* c.G1406C (p.G469A) in FUSCC-14 (**a**) and *BRAF* c.1796_1797insTAC (p.T599dup) in FUSCC-41 (**b**). **c** Representative magnetic resonance imaging (MRI) image showing MTC tumor foci (yellow arrow) and cutaneous

neurofibromas (white arrows) in a patient (FUSCC-24) diagnosed with neurofibromatosis type 1. **d** Representative MRI image showing malignant peripheral nerve sheath tumor (orange arrow) in a patient (FUSCC-24) diagnosed with neurofibromatosis type 1. **e** Sanger sequencing chromatogram showing somatic *NF1* c.3338delT (p.L1113*) in FUSCC-24. **f** Diagram depicting the truncating mutation *NF1* p.L1113* in FUSCC-24 and its downstream impact on cognate mRNA and protein.

Figure S4

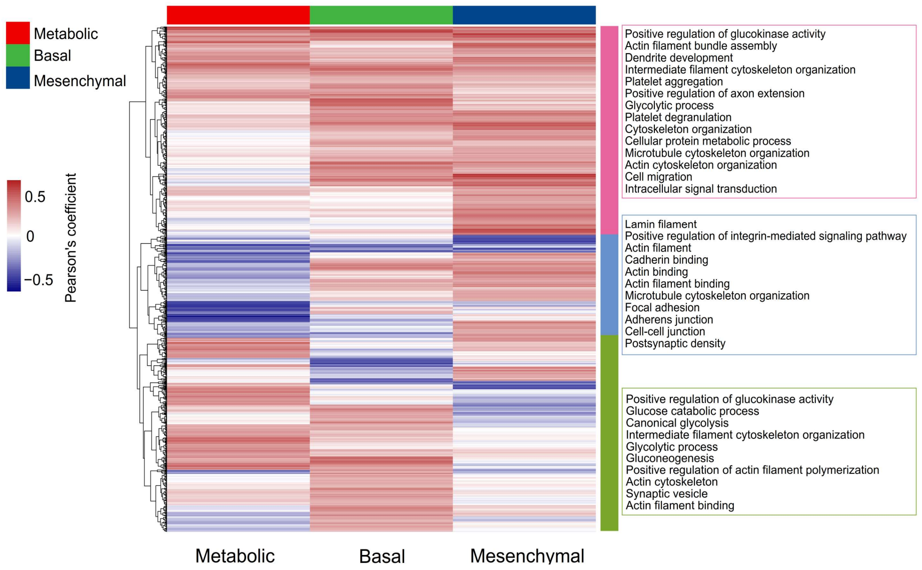

Supplementary Fig. S4 Phosphosite-to-Protein Co-variation Analysis, Related to Fig. 3.

Heatmap showing the results of phosphosite-to-protein co-variation analyses. Pearson's correlation coefficients were computed between phosphosite and cognate protein abundances in the three proteomic subtypes, respectively. A total of 980 significantly co-regulated phosphosite-to-protein pairs (Pearson's coefficient > 0.2, and p-value < 0.05) were used to perform Gene Ontology (GO) pathway enrichment analysis. Representative GO terms were annotated.

Figure S5

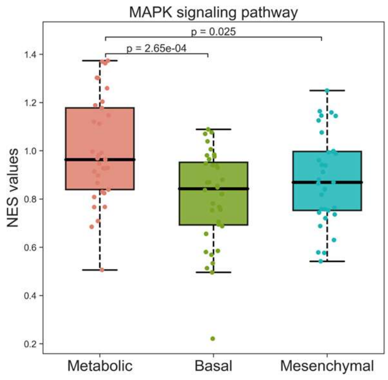

Supplementary Fig. S5 MAPK Signaling Activities in the MTC Proteomic Subtypes, Related to Fig. 4.

Boxplot showing MAPK signaling activities of samples in the three proteomic subtypes. The MAPK signaling activity of each sample was calculated by NES values from KEGG pathway enrichment analysis based on ssGSEA.

Figure S6

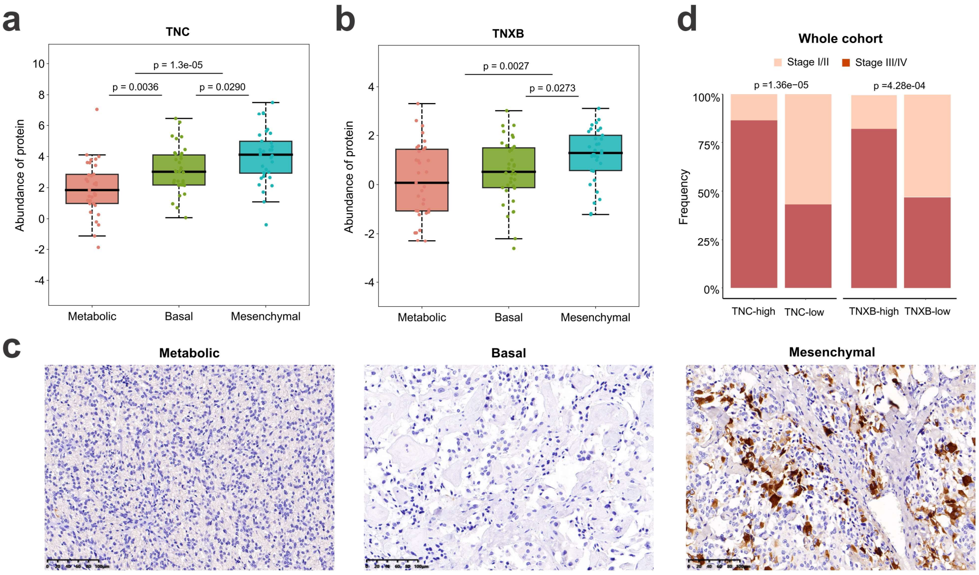

e

Drug	Approved or not	Targets upregulated in Mesenchymal tumors	Phase	Patients	Response	Study
Cabozantinib	Approved by FDA	VEGFR1, PDGFRB	III	330	ORR: 28%; Significantly improved PFS	Elisei et al., 2013
Anlotinib	Approved in mainland China	VEGFR1, PDGFRB	II	91	ORR: 48.4%; DCR: 89%	Li et al., 2021
Surufatinib	Not yet	VEGFR1, CSF1R	II	27	ORR: 22%; DCR: 89%	Chen et al., 2020
Sorafenib	Not yet	VEGFR1, PDGFRB	II	16	ORR: 6.3%; DCR: 94%	Lam et al., 2010
Dovitinib	Not yet	VEGFR1, PDGFRB	II	12	Not listed in detail	Lim et al., 2015
Lenvatinib	Not yet	VEGFR1, PDGFRB	II	59	ORR: 36%, DCR: 80%	Schlumberger et al., 2016
Motesanib	Not yet	VEGFR1, PDGFRB	II	91	ORR: 2%; DCR: 84%	Schlumberger et al., 2009
Pazopanib	Not yet	VEGFR1, PDGFRB	II	35	ORR: 14%; DCR: 71%	Bible et al., 2014
Sunitinib	Not yet	VEGFR1, PDGFRB, CSF1R	II	26	ORR: 38%; DCR: 88%	Ravaud et al., 2017
Axitinib	Not yet	VEGFR1, PDGFRB	II	6	ORR: 0%, DCR: 83%	Locati et al., 2014

Supplementary Fig. S6 Clinical Relevance of Tenascins and Differential Abundance of TKI Targets' Ligands Across the Proteomic Subtypes, Related to Fig. 6.

a, b Histogram comparing the proteomic abundance of TNC (**a**) and TNXB (**b**) across the three proteomic subtypes. *P* values are calculated with Student's t test. For the boxplots: line in the box indicates the median; box borders correspond with the first and third quartiles (25th and 75th percentiles); whiskers extend 1.5 times the interquartile range; outlier data are shown as dots. **c** Representative images (Magnification: 200X) of TNC immunohistochemistry staining on tumor samples belonging to the three proteomic subtypes. Left panel: Metabolic subtype; Middle panel: Basal subtype; Right panel: Mesenchymal subtype. **d** Distribution of TNM staging in TNC-high or TNC-low tumors or in TNXB-high or TNXB-low tumors in the whole cohort. Median abundance is used as the cutoff value for high and low expression and p-values are calculated with Fisher's

exact test. **e** Summary of previous studies utilizing multi-targeted TKIs to treat advanced MTCs in clinical trials, including cabozantinib [6], anlotinib [7], surufatinib [8], sorafenib [9], dovitinib [10], lenvatinib [11], motesanib [12], pazopanib [13], sunitinib [14] and axitinib [15]. Abbreviations: ORR = Objective Response Rate; DCR = Disease Control Rate; PFS = Progression-Free Survival.

References

1 Li, C. *et al.* Integrated Omics of Metastatic Colorectal Cancer. *Cancer Cell* **38**, 734-747.e739, (2020).

2 Silva, J. C., Gorenstein, M. V., Li, G. Z., Vissers, J. P. & Geromanos, S. J. Absolute quantification of proteins by LCMSE: a virtue of parallel MS acquisition. *Mol Cell Proteomics* **5**, 144-156, (2006).

3 Vogel, C. & Marcotte, E. M. Calculating absolute and relative protein abundance from mass spectrometry-based protein expression data. *Nat Protoc* **3**, 1444-1451, (2008).

4 Ishihama, Y. *et al.* Exponentially modified protein abundance index (emPAI) for estimation of absolute protein amount in proteomics by the number of sequenced peptides per protein. *Mol Cell Proteomics* **4**, 1265-1272, (2005).

5 Malmström, J. *et al.* Proteome-wide cellular protein concentrations of the human pathogen Leptospira interrogans. *Nature* **460**, 762-765, (2009).

6 Elisei, R. *et al.* Cabozantinib in progressive medullary thyroid cancer. *J Clin Oncol* **31**, 3639-3646, (2013).

7 Li, D. *et al.* Anlotinib in Locally Advanced or Metastatic Medullary Thyroid Carcinoma: A Randomized, Double-Blind Phase IIB Trial. *Clin Cancer Res* **27**, 3567-3575, (2021).

8 Chen, J. *et al.* Surufatinib in Chinese Patients with Locally Advanced or Metastatic Differentiated Thyroid Cancer and Medullary Thyroid Cancer: A Multicenter, Open-Label, Phase II Trial. *Thyroid* **30**, 1245-1253, (2020).

9 Lam, E. T. *et al.* Phase II clinical trial of sorafenib in metastatic medullary thyroid cancer. *J Clin Oncol* **28**, 2323-2330, (2010).

10 Lim, S. M. *et al.* An open label, multicenter, phase II study of dovitinib in advanced thyroid cancer. *Eur J Cancer* **51**, 1588-1595, (2015).

11 Schlumberger, M. *et al.* A Phase II Trial of the Multitargeted Tyrosine Kinase Inhibitor Lenvatinib (E7080) in Advanced Medullary Thyroid Cancer. *Clin Cancer Res* **22**, 44-53, (2016).

12 Schlumberger, M. J. *et al.* Phase II study of safety and efficacy of motesanib in patients with progressive or symptomatic, advanced or metastatic medullary thyroid cancer. *J Clin Oncol* **27**, 3794-3801, (2009).

13 Bible, K. C. *et al.* A multicenter phase 2 trial of pazopanib in metastatic and progressive medullary thyroid carcinoma: MC057H. *J Clin Endocrinol Metab* **99**, 1687-1693, (2014).

14 Ravaud, A. *et al.* A multicenter phase II study of sunitinib in patients with locally advanced or metastatic differentiated, anaplastic or medullary thyroid carcinomas: mature data from

the THYSU study. *Eur J Cancer* **76**, 110-117, (2017).
15 Locati, L. D. *et al.* Treatment of advanced thyroid cancer with axitinib: Phase 2 study with pharmacokinetic/pharmacodynamic and quality-of-life assessments. *Cancer* **120**, 2694-2703, (2014).
